# ADP-ribose is a competitive inhibitor of methanol dehydrogenases from *Bacillus methanolicus*

**DOI:** 10.1016/j.jbc.2025.110599

**Published:** 2025-08-14

**Authors:** Bao-Di Ma, Jia-Yi Li, Jian-He Xu, Tao Yu, Xu-Dong Kong

**Affiliations:** 1 State Key Laboratory of Microbial Metabolism, School of Life Sciences & Biotechnology, and Zhangjiang Institute for Advanced Study, Shanghai Jiao Tong University, Shanghai, China; 2 School of Chemical and Environmental Engineering, Shanghai Institute of Technology, Shanghai, China; 3 State Key Laboratory of Bioreactor Engineering, Shanghai Collaborative Innovation Center for Biomanufacturing, East China University of Science and Technology, Shanghai, China; 4 Shenzhen Key Laboratory for the Intelligent Microbial Manufacturing of Medicines, Key Laboratory of Quantitative Synthetic Biology, Center for Synthetic Biochemistry, Shenzhen Institute of Synthetic Biology, Shenzhen Institute of Advanced Technology, Chinese Academy of Sciences, Shenzhen, China

**Keywords:** methanol dehydrogenase, activator protein, ADP-ribose, activation mechanism, NAD^+^ hydrolysis

## Abstract

Methanol dehydrogenase (MDH), a representative of Type III alcohol dehydrogenases (ADHs), plays a pivotal role in methanol assimilation pathways, making it a key enzyme for the biosynthesis of chemicals and fuels from one-carbon feedstocks. An activator protein belonging to the Nudix hydrolase family, ACT, was found to increase the activity of MDH by 40-fold. Despite the widespread observation of this *in vitro* activation phenomenon in pairs of type III alcohol dehydrogenases and Nudix hydrolases, the mechanistic details have remained unresolved for decades. Here, we uncover a regulation mechanism in which MDH activation arises from the hydrolytic removal of ADP-ribose (ADPR), a potent inhibitor derived from NAD^+^ degradation, by the ADPRase activity of ACT. This discovery challenges the previously proposed ‘activation’ models, revealing that ACT-mediated ADPR clearance disinhibits MDH rather than directly enhancing catalysis. By combining crystallographic analysis, kinetics, and inhibition assays, we demonstrate that ADPR inhibits MDHs with submicromolar *K*_i_ values, highlighting its potential regulatory role in metabolic networks. Our findings redefine the widespread ‘activation’ of type III ADHs, providing valuable insights into alcohol metabolism and new directions for engineering synthetic methanol utilization pathways.

Methanol, due to its abundance, low cost, and potential for sustainable synthesis from CO_2_ and renewable energy, has become a promising substrate for biosynthesis of chemicals and fuels (1, 2, 3, 4, 5, 6). In recent years, there has been a growing global push toward sustainable chemical production, driven by the need to reduce reliance on fossil resources and mitigate environmental impacts (3, 4, 5). Our research on methanol assimilation mechanisms directly contributes to this shift by advancing more efficient and sustainable biocatalytic processes for industrial biotechnology.

In synthetic methylotrophs, the oxidation of methanol to formaldehyde represents the initial step in methanol assimilation pathways. This reaction requires a highly active and energy-efficient methanol oxidase to enable economically viable bulk chemical production. NAD-dependent methanol dehydrogenases (MDHs) are particularly well-suited for this transformation, as they not only catalyze methanol oxidation but also generate NADH, which serves as both a key reducing agent and a substrate for ATP production through oxidative phosphorylation (3, 7, 8, 9). Notably, NAD-dependent MDHs from *Bacillus methanolicus* MGA-3 (*Bm*MDH2) and *Cupriavidus necator* N-1 (*Cn*MDH2 CT4-1) have been successfully expressed in *Escherichia coli*, enabling the development of synthetic methylotrophs that can grow exclusively on methanol (3, 10).

NAD-dependent MDHs belong to the type III alcohol dehydrogenase (ADH) family, which is evolutionarily distinct from other ADH types such as the zinc-containing medium-chain dehydrogenases (type I) and the metal-independent short-chain dehydrogenases (type II) (11, 12). Although termed ‘iron-containing,’ type III ADHs can bind other metals, such as zinc, cobalt, or manganese (13). In 1991, Dijkhuizen and coworkers discovered that the endogenous activator protein ACT increases the activity of *Bm*MDH1, the first reported NAD-dependent MDH, by 40-fold (14, 15). Subsequent research identified ACT as a member of the Nudix hydrolase family, which catalyzes the hydrolysis of nucleoside diphosphates linked to various moieties (16). A mechanism was proposed in 2002 by Dijkhuizen *et al.*, suggesting that ACT hydrolytically removes the nicotinamide mononucleotide (NMN) moiety from tightly bound NAD^+^ in *Bm*MDH1, thereby enabling a direct electron transfer pathway (17, 18).

Despite extensive investigations into ACT’s role, the precise mechanism by which it activates MDHs remains elusive, with conflicting findings complicating our understanding (7, 13, 15, 16, 17, 18, 19, 20, 21, 22, 23, 24). Contradictory evidence has emerged, including observations that ACT activation effects are absent under certain conditions or in specific reaction directions, such as the reverse reduction of aldehydes to alcohols (15, 19, 21). Additionally, studies have shown that ACT activation is widespread among type III ADHs and is not limited to MDHs, further complicating the mechanistic understanding of these enzymes (19, 20, 21). This lack of clarity has hindered protein engineering efforts aimed at designing artificial methanol assimilation pathways (25, 26, 27).

In contrast to earlier studies primarily focused on *Bm*MDH1 (14, 15, 16, 17, 18), a homolog with moderate activity, our work centers on *Bm*MDH2, a paralog of *Bm*MDH1 that exhibits significantly higher catalytic efficiency and retains the ability to be activated by ACT (19). In this study, we investigated the activation of *Bm*MDH2 by ACT and uncovered a regulation mechanism of MDH where ACT removes adenosine diphosphate ribose (ADPR)—a potent inhibitor formed during NAD^+^ hydrolysis—that restores MDH activity. By integrating crystallographic analysis, catalysis kinetics data, and inhibition assays, we demonstrate that ACT-mediated hydrolysis of ADPR restores MDH activity, thereby resolving the “pseudo-activation” phenomenon. This discovery not only elucidates the activation mechanism of Nudix hydrolases in type III ADHs but also paves the way for engineering enhanced synthetic methylotrophs and optimizing biocatalysts for sustainable industrial applications.

## Results

### Initial characterization of *Bm*MDH2 activation

**Figure 1:**
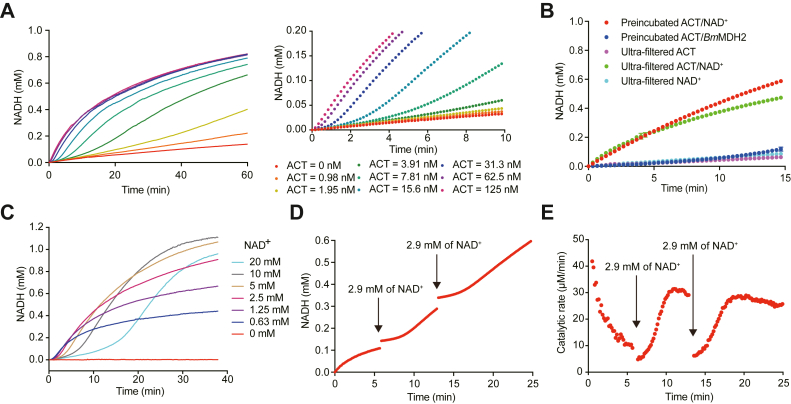
**ACT-catalyzed conversion of NAD^+^ into a compound that activates *Bm*MDH2.***A*, effect of ACT concentration on *Bm*MDH2 activation. The formation of NADH was monitored at 340 nm, with 100 nM *Bm*MDH2 in glycine-NaOH buffer (pH 9.5) containing 0 to 125 nM ACT, 3 mM NAD^+^, 20 mM ethanol, 5 mM MgCl_2_ and 0.5 mM MnCl_2_. Data for the initial 10 min are highlighted in the right panel. Data were from a single experiment. *B*, effects of the following treatments on *Bm*MDH2 activation: preincubated ACT & NAD^+^ (*red*), preincubated ACT & *Bm*MDH2 (*blue*), ultrafiltrate of preincubated ACT & NAD^+^ (*green*), and ultrafiltrate of ACT (*purple*) or NAD^+^ (*cyan*) solutions. Preincubations were performed in glycine-NaOH buffer (pH 9.5) with 5 mM MgCl_2_ and 0.5 mM MnCl_2_ at 25 °C for 30 min. Ethanol, *Bm*MDH2, or NAD^+^ was subsequently added to achieve final concentrations of 10 nM ACT, 20 mM ethanol, 3 mM NAD^+^, and 200 nM *Bm*MDH2. For ultrafiltration, preincubated samples were filtered (10 kDa cutoff, Millipore), and the flowthrough was used for activation assays. Data represent means ± SD (n = 3). *C*, effect of NAD^+^ concentration on *Bm*MDH2 activation. Activity was measured using 100 nM *Bm*MDH2 in glycine-NaOH buffer (pH 9.5) with 50 nM ACT, 0 to 20 mM NAD^+^, 5 mM MgCl_2_, and 0.5 mM MnCl_2_. Data were from a single experiment. *D*, time-course of *Bm*MDH2 activation. Reaction were performed in 200 μl glycine-NaOH buffer (100 mM, pH 9.5) with 5 mM MgCl_2_, 0.5 mM MnCl_2_, 0.25 mM NAD^+^, 0.1 μM *Bm*MDH2, 20 mM ethanol, and 50 nM ACT. Fresh NAD^+^ (4 μl, 150 mM) was added twice during the reaction. Data were from a single experiment. *E*, catalytic rates of *Bm*MDH2 were calculated from the slopes of the data in panel D. Additional replicates of the experiments shown in panels A, C, and D are provided in Fig. S4.

To investigate the activation mechanism of *Bm*MDH2, we assessed the effect of ACT concentration on its activation rate. Previous studies have shown that 0.5 μg/ml ACT (24 pM) was required to maximally stimulate 1.0 μg/ml (25 pM) *Bm*MDH1 *in vitro* (16). In this study, we monitored NADH formation kinetics using 100 nM *Bm*MDH2 and varying ACT concentrations (0–125 nM). High ACT concentrations (>62 nM) triggered immediate *Bm*MDH2 activation, whereas lower concentrations caused delayed full activation (Fig. 1*A*). Analysis of catalytic rates over time (Fig. S1*A*) revealed a linear correlation between the reciprocal of activation time (*i.e.*, activation rate) and ACT concentration (R^2^ = 0.9796, Fig. S1*B*). Specifically, 31.3 nM ACT fully activated 100 nM *Bm*MDH2 within 2.8 min, suggesting that sustained protein‒protein interactions may not be essential for maintaining activation.

To elucidate ACT's catalytic target during MDH activation, we utilized the delay period observed above. Preincubating of ACT with either NAD^+^ or *Bm*MDH2 for 30 min yielded distinct outcomes upon adding the remaining components. ACT preincubated with NAD^+^ eliminated the activation delay, while preincubation with *Bm*MDH2 prolonged the delay (>20 min; Figs. 1*B* and S2). Furthermore, when ACT was removed using a 10 kDa spin filter, the flowthrough retained the activation capability (Fig. 1*B*). These findings lead us to hypothesize that ACT catalyzes the conversion of NAD^+^ to a compound (‘NADx’) responsible for *Bm*MDH2 activation. Notably, the filtered ACT/NAD^+^ mixture lost its activation effect after incubation for 2 h at room temperature, with higher pH accelerating this loss (Supplementary Results).

To further understand the ACT-catalyzed conversion of NAD^+^, the effect of NAD^+^ concentration on the activation rate of *Bm*MDH2 was further evaluated. While a higher NAD^+^ concentration was expected to accelerate the generation of sufficient NADx to stimulate *Bm*MDH2, we observed the opposite: increasing the NAD^+^ concentration prolonged the activation delay (Fig. 1*C*). Analysis of the *Bm*MDH2 catalytic rate over time revealed a strong linear correlation between activation time and NAD^+^ concentration (R^2^ = 0.9952, Fig. S3). Based on these findings, we propose two hypotheses to explain the results described above. 1) ACT Substrate Inhibition: High NAD^+^ concentrations inhibit the ACT-catalyzed conversion of NAD^+^ to NADx, thereby indirectly delaying *Bm*MDH2 activation by reducing NADx availability. 2) Direct NAD^+^ Inhibition: NAD^+^ directly hinders *Bm*MDH2 activation, requiring complete conversion of NAD^+^ into NADx for activation. In this case, NADx functions as a cofactor of *Bm*MDH2.

Based on these hypotheses, we designed experiments to distinguish whether high NAD^+^ concentrations impede *Bm*MDH2 activation *via* ACT-catalyzed NAD^+^ conversion (hypothesis 1) or through direct inhibition of activation (hypothesis 2). Specifically, additional NAD^+^ was introduced to NADx-activated *Bm*MDH2. Under hypothesis 1, since NADx already existed, the reaction rate should remain unaffected. Under hypothesis 2, adding NAD^+^ would immediately decrease the reaction rate, as it would interfere with *Bm*MDH2 activation. In a 200 μl reaction mixture containing 100 nM *Bm*MDH2, 50 nM ACT, 0.25 mM NAD^+^, and 20 mM ethanol, *Bm*MDH2 exhibited full activation with a catalytic rate of 42 μM/min (turnover frequency of 7 s^–1^) from the start of the reaction (Fig. 1*D*). After 5.7 min, an additional NAD^+^ dose (raising the final concentration by 2.9 mM) reduced the catalytic rate to 4.8 μM/min (Fig. 1, *D* and *E*). Following a delay, the catalytic rate recovered to 30.3 μM/min, indicating reactivation. When another NAD^+^ supplement (final concentration increased by 2.9 mM) was added 2 minutes later, the catalytic rate again dropped and subsequently recovered (Fig. 1, *D* and *E*).

These results support the second hypothesis, suggesting that higher NAD^+^ concentrations directly hinder *Bm*MDH2 activation. In summary, this study provides the first experimental evidence demonstrating that *Bm*MDH2 activation is reversible, a feature unexplained by previously reported activation mechanisms (18).

### Discovery of a hidden MDH inhibitor

**Figure 2:**
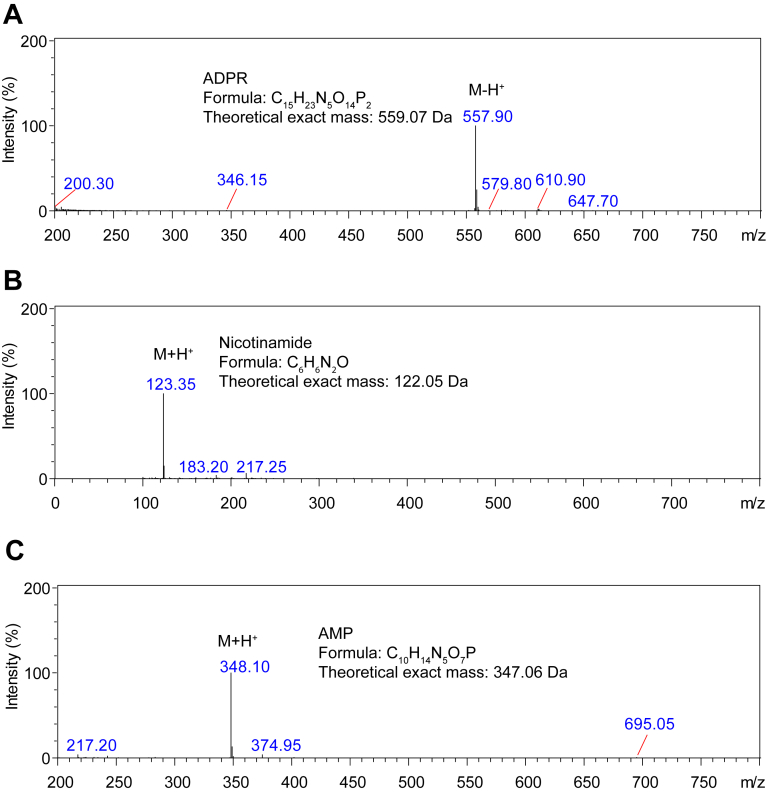
**Identification of ADPR, nicotinamide, and AMP by LC-MS.***A*, mass spectrum of ADPR detected in the NAD^+^ sample. *B*, mass spectrum of nicotinamide detected in the ACT treated NAD^+^ sample. *C*, mass spectrum of AMP detected in the ACT treated NAD^+^ sample. The NAD^+^ (3 mM) were incubated with or without 10 nM ACT in glycine-NaOH buffer (pH 9.5) containing 5 mM MgCl_2_ for 60 min at 25 °C. Samples were analyzed by LC-MS on Shimadzu LCMS-2020. The mass of ADPR (*A*) was identified in the negative ionization mode, while mass of nicotinamide (*B*) and AMP (*C*) were identified in the positive ionization mode.

**Figure 3:**
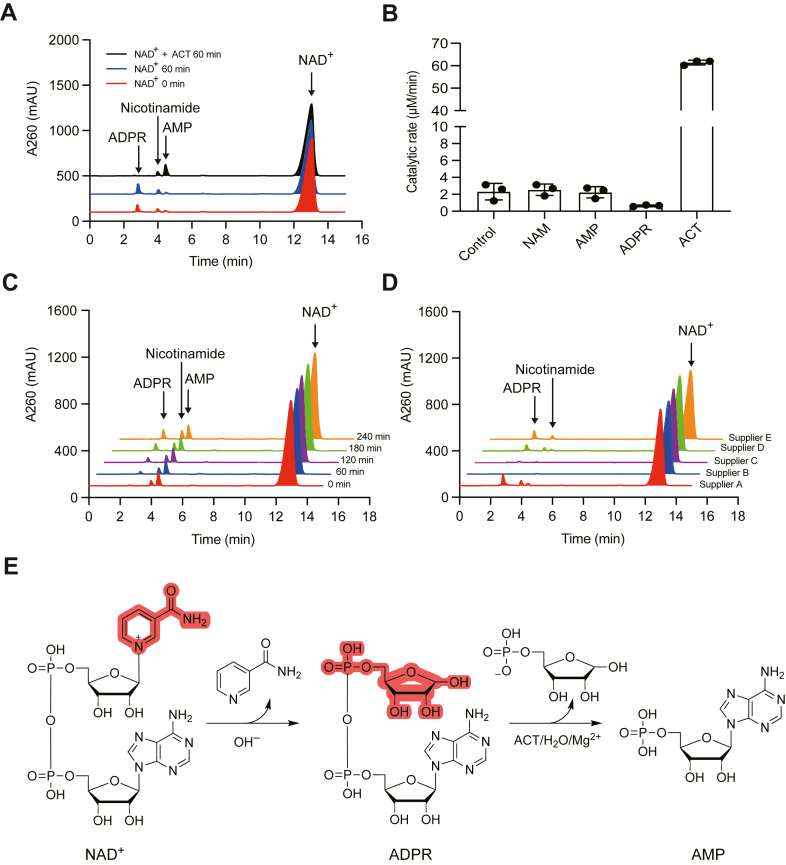
**ACT-catalyzed hydrolysis of ADPR derived from NAD^+^ degradation.***A*, HPLC analysis of NAD^+^ before and after treatment with ACT. NAD^+^ (3 mM) was incubated with (*black*) or without (*blue*) 10 nM ACT in glycine-NaOH buffer (pH 9.5, 5 mM MgCl_2_) at 25 °C for 60 min. Unincubated NAD^+^ (*red*) served as a control. Compounds were identified *via* LC-MS. *B*, effects of NAM, AMP, and ADPR (0.3 mM each) on *Bm*MDH2 activity in the presence of 3 mM NAD^+^ and 40 mM ethanol. Data are presented as means ± SDs (n = 3). *C*, degradation of ACT-treated NAD^+^ in glycine-NaOH buffer (pH 9.5) at 25 °C. ACT was removed by ultrafiltration before analysis. *D*, ADPR content in NAD^+^ from different suppliers. ADPR percentages (mol/mol) were 5.27%, 0.08%, 0.63%, 3.06%, and 4.32% for suppliers *A*, *B*, *C*, *D*, and *E*, respectively. *E*, schematic of the reactions during ACT incubation with NAD^+^. NAD^+^ spontaneously degrades to NAM and ADPR, with ACT catalyzing ADPR hydrolysis in the presence of Mg^2+^.

To investigate the structure of NADx and its interaction with *Bm*MDH2, we analyzed the ACT-treated NAD^+^ sample using high-performance liquid chromatography (HPLC) and liquid chromatography-mass spectrometry (LC-MS) (Fig. 2). LC-MS revealed two additional peaks alongside NAD^+^, AMP, and nicotinamide (NAM), with the AMP peak showing a significant increase compared to untreated NAD^+^. Notably, one peak disappeared, which was identified as ADPR (Figs. 2 and 3*A*).

When NAD^+^ was incubated in glycine-NaOH buffer (pH 9.5) for 60 min, ADPR and NAM increased by about 40%, while the AMP peak remained unchanged (Fig. 3*A*). HPLC analysis of ACT-treated NAD^+^ revealed that ADPR was converted into AMP, with no significant change in NAD^+^ levels (Fig. 3*A*). This hydrolysis of ADPR to AMP aligns with the previously reported ADPRase activity of ACT (16). Specifically, we measured the ADPRase activity of ACT to be 61.2 ± 0.5 U/mg at pH 8.0 and 51.6 ± 0.4 U/mg at pH 9.5. However, NAD^+^ was not completely transformed and an unknown compound that may be represented by NADx did not emerge. This suggests a new mechanism for *Bm*MDH2 activation, beyond previous hypotheses.

To explore whether AMP, NAM, or ADPR affects *Bm*MDH2 activity, we tested their impact at a final concentration of 0.3 mM. Neither AMP nor NAM significantly influenced activation, but ADPR inhibited *Bm*MDH2 activity by 72% (Fig. 3*B*). These results suggest that ADPR inhibition of *Bm*MDH2 is the true cause of the ACT-mediated “activation.” ACT likely eliminates ADPR, an impurity in NAD^+^ products, thereby restoring *Bm*MDH2 activity. The “activation” delay upon NAD^+^ addition can be explained by the concurrent introduction of ADPR from untreated NAD^+^.

We further examined the stability of ACT-treated NAD^+^ at higher pH. As shown in Figure 3*C*, the concentrations of ADPR and NAM increased linearly over time, consistent with the known hydrolysis of NAD^+^ at basic pH (28, 29). The rate constant for NAD^+^ hydrolysis was determined to be 0.011 h^−1^ (*t*_1/2_ = 64 h, Fig. S5). Additionally, the ADPR content in NAD^+^ from different suppliers was found to range from 0.08% to 5.27% (Fig. 3*D*). These findings support the new activation mechanism: ACT hydrolyzes ADPR, a degradation product of NAD^+^, to remove its inhibition of *Bm*MDH2, thereby “activating” the enzyme (Fig. 3*E*).

### ADPR is a potent competitive inhibitor of MDH

**Figure 4:**
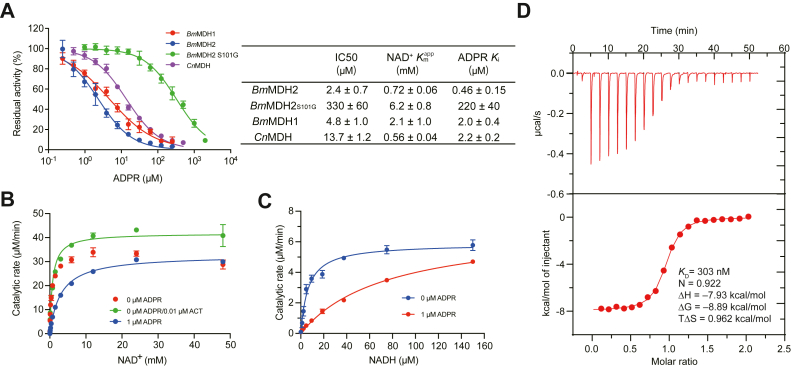
**Characterization of ADPR inhibition of and binding affinity for MDHs.***A*, inhibition of MDHs by ADPR in the presence of 3 mM NAD^+^. Data are shown as means ± SDs (n = 3). Apparent *K*_M_ values for NAD^+^ were determined in the presence of 10 nM ACT (Fig. S6). *B-C*, inhibition kinetics of ADPR on ethanol dehydrogenation (*B*) and formaldehyde reduction (*C*) catalyzed by *Bm*MDH2. Data are presented as means ± SDs (n = 3). *D*, binding affinity of ADPR to *Bm*MDH2 determined by isothermal titration calorimetry (ITC).

We performed detailed quantitative and mechanistic analyses of ADPR's inhibitory effects on MDHs. To eliminate the potential interference of ADPR from impure NAD^+^ or its hydrolysis at high pH, we used ACT to prepare ADPR-free NAD^+^ and conducted inhibition assays at pH 8.0. The residual activities of *Bm*MDH2 and several representative MDHs, including *Bm*MDH1 (12) and the activation-insensitive mutants *Cn*MDH2 CT4-1 (24) and *Bm*MDH2_S101G_, were assessed in the presence of varying ADPR concentrations. As shown in Figure 4*A*, both *Bm*MDH1 and *Bm*MDH2 exhibited inhibition by ADPR, with *IC*_50_ values in the low micromolar range (4.8 ± 1.0 μM and 2.4 ± 0.7 μM, respectively). The *IC*_50_ of *Cn*MDH2 CT4-1 was more than fivefold higher than that of *Bm*MDH2, whereas the *IC*_50_ of *Bm*MDH2_S101G_ was over two orders of magnitude greater. The apparent *K*_M_ values of these MDHs for NAD^+^ were determined in the presence of 10 nM ACT to minimize interference from NAD ^+^ degradation (Figs. 4*A* and S6). The *K*_i_ values for ADPR followed similar trends to the *IC*_50_ values, despite the relatively high *K*_i_ values of *Bm*MDH1 and *Bm*MDH2_S101G_, which is primarily due to their higher apparent *K*_M_ values. These results further explain the activation insensitivity of *Bm*MDH2_S101G_^20^. Notably, the *K*_i_ value of ADPR for *Cn*MDH2 CT4-1 was 4.8-fold greater than that for *Bm*MDH2, consistent with the observation that *Cn*MDH2 CT4-1 is less sensitive to ACT activation (24). Considering the new mechanism of “activation” discovered in this study, it is noteworthy that previously reported data may have been biased by the ADPR content in the NAD^+^ used in activity assays.

We also examined the inhibition kinetics of ADPR with *Bm*MDH2 to gain deeper insights into the underlying mechanism. As shown in Figure 4*B*, kinetic analysis of *Bm*MDH2 showed a substrate inhibition trend, whereas the addition of 10 nM ACT resulted in the data fitting standard Michaelis-Menten kinetics more closely (*K*_M_ = 0.72 ± 0.06 mM, *k*_cat_ = 420 ± 20 min^−1^). The introduction of 1.0 μM ADPR caused a decrease in *k*_cat_, while *K*_M_ remained unchanged. However, the hydrolysis of NAD^+^ could influence the kinetics of *Bm*MDH2, as *Bm*MDH2 exhibits a higher affinity for ADPR than ACT (*K*_M_ = 15.9 μM for ADPR, Fig. S7). Given that NADH is more stable than NAD^+^ (Fig. S8), we assessed the inhibition kinetics of *Bm*MDH2 in the reverse reaction (formaldehyde to methanol). As shown in Figure 4*C*, ADPR greatly increased the *K*_M_ of *Bm*MDH2 from 6.7 ± 1.7 μM to 80 ± 20 μM, with a weaker effect on the *k*_cat_ (59 ± 2 min^−1^ vs. 73 ± 9 min^−1^), indicating that ADPR is a competitive inhibitor. The *K*_i_ value of ADPR derived from the inhibition kinetics data in Figure 4*C* was 0.092 μM, significantly lower than the value calculated from the *IC*_50_ (*K*_i_ = 0.46 ± 0.15 μM). Additionally, isothermal titration calorimetry (ITC) revealed that ADPR binds to *Bm*MDH2 with a binding constant (*K*_D_) of 303 nM (Fig. 4*D*). Together, these findings demonstrate that ADPR inhibits both directions of the oxidoreduction reaction catalyzed by *Bm*MDH2 in a competitive manner with submicromolar affinity.

### Structural insights into ADPR inhibition and hydrolysis

**Figure 5:**
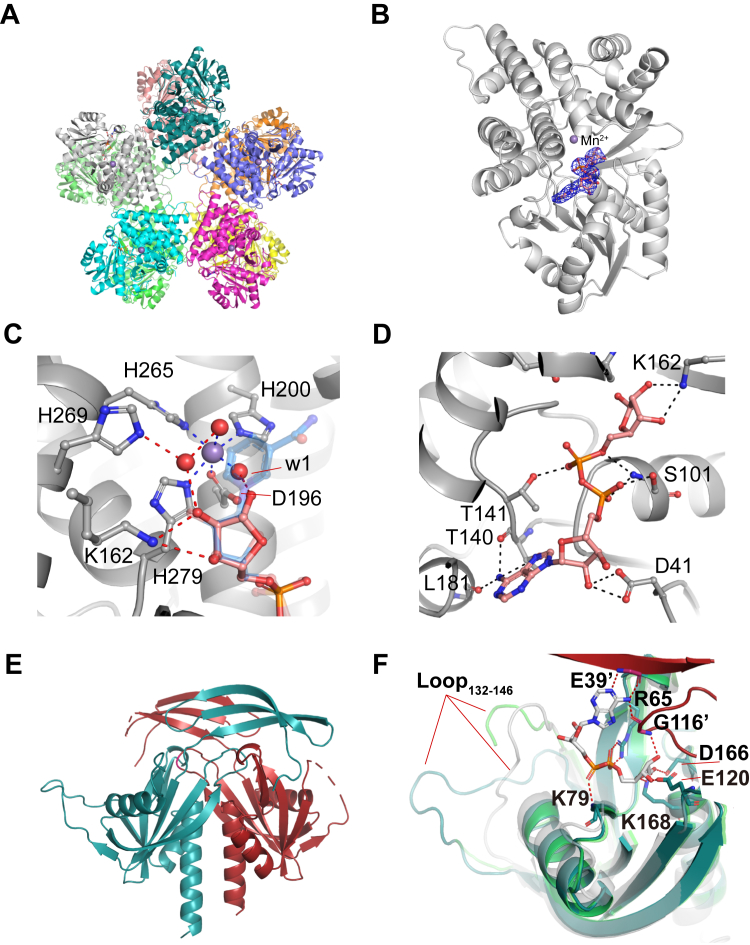
**Structural basis of ADPR inhibition and hydrolysis**. *A*, dDecameric structure of *Bm*MDH2 in the *Bm*MDH2-ADPR complex (2.6 Å resolution). *B*, ADPR and Mn^2+^ binding sites in the *Bm*MDH2 monomer. ADPR and Mn^2+^ are shown as sticks and spheres, respectively. Omit map of ADPR is contoured at *σ* = 1.5. *C*, binding conformations of ADPR and NAD ^+^ at the *Bm*MDH2 catalytic center. Mn^2+^ and water molecules are shown as spheres. ADPR, NAD^+^, and key residues are shown as sticks with carbon atoms in *pink* (ADPR), transparent *blue* (NAD^+^), and *gray* (*Bm*MDH2). Coordinate bonds (*blue* dashed lines) and hydrogen bonds (*red* dashed lines) are highlighted. *D*, hydrogen bond interactions (*black* dashed lines) between ADPR and the Rossmann fold of *Bm*MDH2. ADPR and interacting residues are shown as balls and sticks. *E*, overall structure of the ACT dimer, with monomers colored deep teal and firebrick (PDB ID: 8WV3). *F*, catalytic center of ACT. Structures of ACT (deep teal and firebrick), NDPSase from *Bdellovibrio bacteriovorus* (*gray*, PDB ID: 5C7T) (30), and ACT with an alternative loop_132–146_ conformation (*green*, PDB ID: 9JAX) were superimposed. ADPR from the NDPSase structure (sticks) and ACT residues involved in potential hydrogen bonding (dashed lines) are shown.

To understand how ADPR binds *Bm*MDH2 with greater affinity than NAD^+^ and NADH, we solved the crystal structures of *Bm*MDH2_apo, *Bm*MDH2_ADPR, and *Bm*MDH2_NAD^+^ at resolutions of 2.8 Å, 2.6 Å, and 2.6 Å, respectively. As shown in Figure 5*A*, *Bm*MDH2 adopts a decameric structure, consistent with previous cryo-EM findings (16). Clear electron densities for ADPR and NAD^+^ were observed in their respective complex structures (Fig. 5*B*). At the active center of *Bm*MDH2_apo, Mn^2+^ was coordinated by residues D196, H200, H265, and H279 along with two water molecules (Fig. 5*C*; see Supplementary results for validation of the metal ion).

Both ADPR and NAD^+^ bind in highly consistent conformations, forming 19 and 20 hydrogen bonds, respectively (Figs. 5*C* and S9). The key difference lies in the additional hydrogen bonds of NAD^+^ with T149 and S146, and the interaction between the free hemiacetal hydroxyl group of ADPR and Mn^2+^ *via* water molecule (w1) (Fig. 5*C*). Notably, the NAM ring of NAD^+^ occupies the w1 position during binding, suggesting that the differential effects of w1 on ADPR and NAD^+^ binding contribute to their differing affinities. Additional ITC assays using ADPR and EDTA-treated *Bm*MDH2 showed no binding (Fig. S10), confirming the importance of the metal ion for ADPR binding to *Bm*MDH2. To investigate the metal ion's role in ADPR binding specificity and the generality of ADPR inhibition, we examined the inhibitory effects of ADPR on other dehydrogenases, including one Zn^2+^-dependent type I ADH and four metal-independent type II ADHs. None of these enzymes showed significant inhibition by 0.5 mM ADPR (Fig. S11), supporting the hypothesis that the metal ion is crucial for ADPR binding to *Bm*MDH2. Further structural analysis highlighted the role of the S101 residue in stabilizing the pyrophosphate group of NAD^+^/NADH within the active site (Fig. 5*D*). Mutation of S101 to glycine (*Bm*MDH2_S101G_) disrupted this interaction, reducing binding affinity for both ADPR and NAD^+^, as reflected by increased *K*_i_ and *K*_M_ values (Fig. 4*A*), thereby explaining the mutation's insensitivity to ADPR inhibition.

We also solved the crystal structures of ACT with the flexible loop_132-146_ captured in two conformations, at resolutions of 2.0 and 2.4 Å (Fig. 5, *E* and *F*). ACT forms a homodimer in solution, with the active site composed of residues from both monomers (Fig. 5, *E* and *F*). The overall structure of ACT is similar to that of NDPSase from *Bdellovibrio bacteriovorus* (PDB ID: 5C7T) (30) and *Tt*ADPRase from *Thermus thermophilus* (PDB ID: 1V8L) (31), except for the conformation of the flexible loop, which is crucial for substrate binding (30). Superimposing ACT onto the *Tt*ADPRase-ADPR complex revealed that ADPR interacts with ACT through hydrogen bonds with residues K79, E39′, R65, G116′, K168, E120, and D166 (Fig. 5*F*). Mutational analysis of key residues within ACT’s active site identified R92, E93, and E97 in the Nudix box motif (residues 78∼100) as essential for ADPR hydrolysis (Table S2). These residues are involved in coordinating metal ions (Mg^2+^) and water molecules, consistent with observations in *Tt*ADPRase and *Ec*MutT from *E. coli* (31, 32). In these systems, E93 acts as a general base, E97 facilitates metal binding, and R92 positions E93 for optimal function.

## Discussion

**Figure 6:**
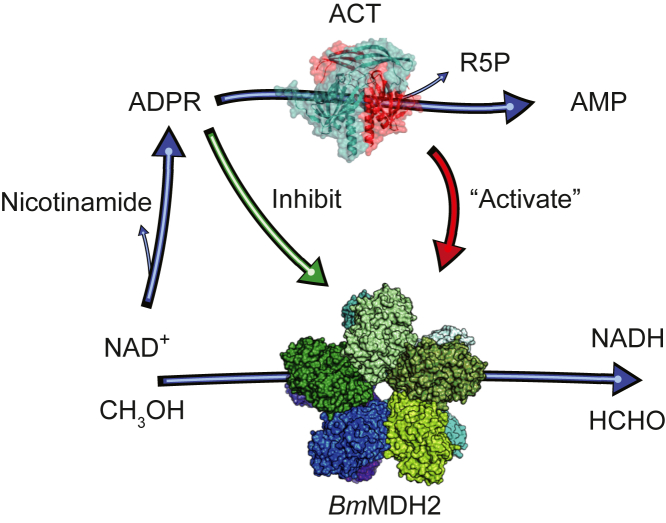
**Schematic representation of ADPR metabolism and its regulatory effects on *Bm*MDH2 activity.***Bm*MDH2 catalyzes the oxidation of methanol (CH_3_OH) to formaldehyde (HCHO) and the reduction of NAD^+^ to NADH. NAD^+^ spontaneously degrades into nicotinamide and ADPR, which inhibits *Bm*MDH2 activity (*green arrow*). ADPR is further hydrolyzed by ACT into AMP and ribose 5-phosphate (R5P) in the presence of Mg^2+^, alleviating its inhibitory effect on *Bm*MDH2. The ‘activation’ and inhibition interplay between ACT and ADPR on *Bm*MDH2 illustrates a regulatory mechanism for maintaining methanol metabolism. Protein structures of ACT and *Bm*MDH2 are shown in surface representation.

The mechanism by which ACT activates NAD-dependent methanol dehydrogenase (MDH) has remained unresolved since its discovery in 1991 (14, 15, 16, 17, 18). Our study reveals that this ‘activation’ results from the enzymatic hydrolysis of adenosine diphosphate ribose (ADPR), a potent MDH inhibitor derived from NAD^+^ degradation (Fig. 6). By resolving this ‘pseudo-activation’ phenomenon, we redefine the regulatory role of Nudix hydrolases in type III alcohol dehydrogenases and expand their implications to broader metabolic contexts.

Our findings demonstrate that ADPR inhibits MDHs with submicromolar *K*_i_ values, significantly suppressing their activity. While ADPR has been studied for its role in intracellular signaling (33, 34), its strong inhibitory effects on enzymes involved in central metabolism, such as MDHs, have largely been overlooked. This discovery highlights the potential regulatory significance of ADPR in metabolic pathways across diverse organisms (35, 36, 37, 38). Notably, ADPR concentrations *in vivo* can vary significantly, ranging from 5 to 73 μM in human cells (35, 36), to 105 μM in yeast (37), and up to 800 μM in some bacterial species (38). This suggests that ADPR accumulation might play an important role in modulating the activity of type III ADHs, with Nudix hydrolases acting as key players in maintaining metabolic homeostasis by counteracting ADPR accumulation (39, 40). A recent study highlights the role of NAD^+^/ADPR cycling in bacterial immunity (41). This aligns with our findings, as both studies reveal the critical impact of ADPR accumulation on cellular processes and underscore the importance of maintaining NAD^+^/ADPR balance for metabolic homeostasis.

The structural analysis of ADPR binding to MDHs revealed that ADPR interacts with the catalytic site more strongly than NAD^+^, explaining the high sensitivity of MDHs to ADPR inhibition. This insight opens new avenues for engineering ADPR-insensitive MDH variants, such as *Bm*MDH2_S101G_ (20, 24), to improve biocatalysis efficiency in synthetic methylotrophic pathways.

This work has significant implications for synthetic biology. By elucidating the mechanistic basis of MDH activation, our study provides a foundation for designing more efficient methylotrophic strains for sustainable methanol-based production (1, 2, 3, 4, 5, 6). Additionally, our findings highlight the need for more stringent experimental controls in previous studies, as ADPR contamination in NAD^+^ preparations could have influenced past research on type III ADHs.

In summary, this study resolves a 30-year mystery in MDH activation, revealing that ACT-mediated activation is, in fact, a relief from ADPR inhibition. Beyond its impact on MDHs, our findings expand the understanding of ADPR’s role in regulating enzymatic activity in central metabolism and provide new insights for protein engineering and metabolic pathway optimization in biomanufacturing.

## Experimental procedures

### General materials

PrimSTAR Max DNA Polymerase was purchased from Takara Co., Ltd. Plasmid mini preparation kit, PCR purification kit, and other molecular biology reagents were obtained from Beyotime Biotechnology Inc. Primer synthesis and DNA sequencing were conducted by Sangon Biotechnology Co., Ltd (Shanghai, China). NADH, AMP, ADPR, ethanol, methanol, formaldehyde, and other chemicals were obtained from standard commercial sources. NAD^+^ sodium from different suppliers, including Aladdin (CAT#N111610, CAT#B132541), Sigma (CAT#N7004), TCI (CAT#D0919), and J&K (CAT#341773), was used in this study. Protein crystallization kits were purchased from Hampton Research. Polyethylene glycol 3350 (PEG 3350), polyethylene glycol 400 (PEG 400), sodium citrate tribasic hydrate, and MES monohydrate used in protein crystallization were purchased from Sigma-Aldrich Trading Co., Ltd. Sodium formate and sodium acetate were purchased from Alfa Aesar Chemical Co., Ltd. Bis-Tris propane was obtained from Aladdin Bio-Chem Technology Co., Ltd.

### The cloning, expression, and purification of enzymes

The codon-optimized genes of activator protein ACT (GenBank accession: EIJ83380.1), *Bm*MDH1 (GenBank accession: EIJ77596.1), and *Bm*MDH2 (GenBank accession: EIJ83020.1) from *B. methanolicus* MGA3 were synthesized by Genewiz Biotech Co., Ltd (Suzhou, China). The ORF sequences of genes and the corresponding amino acid sequences are listed in the Supporting Information. These genes were cloned into the pET21a(+) between *Nde*I and *Xho*I restriction sites. The *Cn*MDH2 CT4-1 mutant (A26V, A31V, and A169V) from *C. necator* N-1 was cloned into pET28a(+) using *Nco*I and *Xho*I restriction sites. Variants of *Bm*MDH2 and ACT were constructed following the standard procedure of site-directed mutagenesis using primers as listed in Table S1.

The recombinant plasmids containing the genes of ACT, *Bm*MDH1, *Bm*MDH2, or their variants were transformed into *E. coli* BL21(DE3) for recombinant expression. The cells were grown in 600 ml of lysogeny broth (LB) medium containing 100 μg/ml ampicillin in 2.5 L flasks (200 rpm) at 37 °C until the OD_600_ reached 0.8 to 1.0. The expression was induced by 0.1 mM isopropyl β-D-1-thiogalactopyranoside (IPTG) at 16 °C for 20 h. The recombinant pET28(a)-*cnmdh2 CT4-1* plasmid was transformed into *E. coli* Rosetta(DE3) for expression. The cells were cultured in LB medium containing 50 μg/ml kanamycin and induced by 0.2 mM IPTG at 25 °C for 12 h.

The cells were harvested by centrifugation and resuspended in buffer A (20 mM potassium phosphate, pH 7.5, 500 mM NaCl, and 10 mM imidazole). The obtained cell suspension was lysed by ultrasonication for 30 min while maintained on ice. The cell lysate was centrifuged at 30,700×*g* at 4 °C for 30 min to remove the cell debris. After loading the supernatant onto a Ni-affinity column and washing with buffer A, 5 to 100% gradients of buffer B (20 mM potassium phosphate, pH 7.5, 500 mM NaCl, 500 mM imidazole) were used for elution. As a next step, the protein for crystallization was purified using size-exclusion chromatography (Superdex 75 10/300 Gl for ACT and Superdex 200 10/300 for MDHs) in a buffer containing 20 mM Tris-HCl (pH 7.5) and 150 mM NaCl. Fractions containing pure protein were pooled and concentrated with an Amicon Ultra-15 spin filter (molecular weight cut off of 10 kDa) for protein crystallization.

### The effect of ACT and NAD^+^ on the catalytic rate of *Bm*MDH2

The catalytic processes of MDHs were monitored by measuring NADH absorbance at 340 nm using a Tecan SPARK UV-Vis plate reader (Tecan Austria GmbH) at 25 °C. The assays evaluating the effect of ACT concentration on *Bm*MDH2 catalytic rate were performed in 200 μl of glycine-NaOH buffer (100 mM, pH 9.5) containing 5 mM MgCl_2_, 3 mM NAD^+^, 100 nM *Bm*MDH2, 0.5 mM MnCl_2_, 20 mM ethanol, and 0 to 125 nM of ACT in a 96-well plate. To investigate the effect of NAD^+^ concentration on the activation rate of 100 nM *Bm*MDH2, ACT concentration was fixed at 50 nM, and NAD^+^ concentration was varied from 0 to 20 mM.

### The activation effect of pre-incubated ACT/*Bm*MDH2 and ACT/NAD^+^

ACT (12.5 nM) was pre-incubated with 3.75 mM of NAD^+^ or 250 nM of *Bm*MDH2, respectively, in glycine-NaOH buffer (100 mM, pH 9.5) containing 5 mM MgCl_2_ at 25 °C. After 30 min of incubation, the remaining components were added to establish a final reaction system containing 10 nM ACT, 5 mM MgCl_2_, 3 mM NAD^+^, 200 nM *Bm*MDH2, 0.5 mM MnCl_2,_ and 20 mM ethanol. The NADH concentration was monitored by measuring the 340 nm absorbance at 25 °C. ACT was further removed using an Amicon Ultra-15 spin filter from the above-mentioned pre-incubated ACT/NAD^+^ mixture (molecular weight cut off of 10 kDa). Before the measurement of *Bm*MDH2 activity, the remaining components were added to the flow-through solution to establish a reaction system consisting of 5 mM MgCl_2_, 3 mM NAD^+^, 200 nM *Bm*MDH2, 0.5 mM MnCl_2,_ and 20 mM ethanol. Control assays were conducted using pre-incubated ACT or NAD^+^ alone, filtered through the Amicon Ultra-15 spin filter.

### The HPLC and LC-MS analysis

The HPLC analysis was performed on the Agilent 1260 Infinity II equipped with an Agilent Infinity Lab Poroshell 120 EC-C18 column (4.6 mm × 50 mm, 2.7 μm) under UV detection at 260 nm. As a mobile phase, water containing 0.1% (v/v) formic acid was used at a flow rate of 1 ml/min to analyze NAD^+^. NADH was analyzed using a mobile phase consisting of 0.1% (v/v) formic acid in water (A) and methanol (B) with a flow rate of 1 ml/min. The elution program consisted of 15 min of 100% A followed by 10 minutes of 95% A & 5% B. The mass of compounds was identified by electrospray ionization mass spectrometry (ESI-MS) in both positive and negative ion mode on a single quadrupole liquid chromatography mass spectrometer (LC-MS/2020, Shimadzu).

### The preparation of pure NAD^+^ without ADPR

The NAD^+^ at 200 mM was incubated with 1 μM of ACT for 60 min in Tris-HCl buffer (100 mM, pH 8.0) containing 5 mM MgCl_2_. Following the incubation, NAD^+^ was precipitated from the reaction mixture by adding three volumes of ethanol. After centrifugation at 10,000×*g* and 4 °C for 10 min, the precipitate containing pure NAD^+^ was dissolved in water and subsequently filtered through the Amicon Ultra-15 spin filter to remove the residual ACT.

### The inhibition assay of MDHs

Inhibition constants (*K*_i_) were determined by measuring the residual activities of MDHs in the presence of different dilutions of ADPR (2-fold dilutions, ranging from 0 μM to 2000 μM final concentration) using ethanol as substrate. Activities were measured at 25 °C in 200 μl Tris-HCl buffer (100 mM, pH 8.0) containing 40 mM ethanol, 3 mM purified NAD^+^ (without ADPR and ACT), 0.5 mM MnCl_2_ and adequate MDHs by monitoring the change in 340 nm absorption over 30 min using a Tecan SPARK plate reader. The rate of absorbance change is proportional to enzyme activity. The *IC*_50_ values were determined by fitting sigmoidal curves to the data using the following four-parameter logistic equation:

$$Y=\frac{100}{1+10^{\left({\text{LogIC}}_{50}-X\right)p}}$$

Wherein Y is the residual activity (%) of MDHs, X is the logarithm of ADPR concentration, *IC*_50_ is the concentration of inhibitor that produces 50% inhibition, and p is the Hill coefficient. The *K*_i_ values were calculated based on the *IC*_50_s using the Cheng-Prusoff equation:

$$K_{i}=\frac{{IC}_{50}}{1+\frac{{\left[S\right]}_{0}}{K_{M}}}$$

Wherein [S]_0_ is the initial concentration of NAD^+^ used for inhibition assay, and *K*_M_ is the Michaelis-Menten constant of the MDHs for NAD^+^ determined as described below.

### The inhibition effect of ADPR on type I and type II alcohol dehydrogenases

The type I alcohol dehydrogenases, ADH from *Saccharomyces cerevisiae*, was purchased from TCI (CAT# A0200). Four metal ion-independent type II alcohol dehydrogenases, isopropanol dehydrogenase from *Brucella suis* (IPADH_M4_) (42), *Cb*FDH from *Candida boidinii* (43), glucose dehydrogenase (*Bm*GDH) from *Bacillus megaterium* IWG3 (44), and *Ba*AlaDH from *Bacillaceae* (45) were expressed as described in references, and cell-free extracts were used in the activity assay. The activity of IPADH_M4_ was measured in Tris-HCl buffer (100 mM, pH 8.0) with 800 mM isopropanol as the substrate. The enzymatic activities of *Cb*FDH, *Bm*GDH, and *Ba*AlaDH were measured in HEPES buffer (100 mM, pH 7.5) using 50 mM ammonium formate, glucose, and alanine as substrates, respectively (46). The activity of ADH from *S. cerevisiae* was measured in Tris-HCl buffer (100 mM, pH 8.8) with 40 mM ethanol as the substrate. The commercial NAD^+^ was used as a coenzyme in the assays at a final concentration of 0.5 mM. ACT was included at a final concentration of 1 μM to investigate its activation effect on various dehydrogenases. The ADPR was added at concentrations of 0.5 or 3 mM to evaluate the inhibitory effect of ADPR on the activity of different dehydrogenases.

### The kinetic analysis of MDHs and ACT

The kinetic assays for MDHs were conducted in 200 μl of Tris-HCl buffer (100 mM, pH 8.0) at 25 °C. NAD^+^ and NADH used for the kinetic assays were pretreated with ACT as described above. The reaction mixtures for the ethanol dehydrogenation contained 40 mM ethanol, adequate MDHs, and various concentrations of NAD^+^ (ranging from 0 to 48 mM). Enzymatic catalytic rates were determined by monitoring the increase in NADH absorbance at 340 nm. To determine the kinetics of *Bm*MDH2 for the reverse direction (formaldehyde reduction), the reaction mixtures contained 1 mM DTT, 100 nM *Bm*MDH2, 20 mM formaldehyde, 0.5 mM MnCl_2,_ and 0 to 150 μM of NADH. The catalytic rates of formaldehyde reduction were measured by monitoring the decrease of NADH fluorescence, using excitation and emission wavelengths of 340 nm and 535 nm, respectively. To prevent the accumulation of ADPR during the kinetic assay, 10 nM ACT and 5 mM MgCl_2_ were added to the reaction mixtures.

The kinetic assays for ACT were conducted in 100 μl of Tris-HCl buffer (100 mM, pH 8.0) containing 5 mM MgCl_2_, 0.5 nM ACT and various concentrations of ADPR (ranging from 0 to 400 μM). The reaction was terminated by adding 10 μl of 3 M H_2_SO_4_ after incubation at 25 °C for 5 min, followed by analysis using HPLC. The *k*_cat_ and *K*_M_ values were determined by fitting the initial velocity *versus* substrate concentration data to the Michaelis-Menten equation using GraphPad Prism. One unit (U) of ACT activity is defined as the amount of enzyme that catalyzes the conversion of 1 μmol ADPR per minute under conditions described above.

### The isothermal titration calorimetry (ITC) assay

The ITC assay was performed on a Malvern Microcal PEAQ-ITC instrument (Malvern Panalytical), at 25 °C with stirring of 750 rpm. The sample cell was filled with 200 μl of protein solution (30 μM purified *Bm*MDH2 in 200 mM Tris-HCl, pH 8.0, 0.5 mM MnCl_2_). The 300 μM ADPR solution containing 200 mM Tris-HCl (pH 8.0) and 0.5 mM MnCl_2_ was titrated at a rate of 0.5 μl/s at 150 s time intervals (first injection of 0.4 μl followed by 19 injections of 2 μl). The titration data were analyzed using MicroCal PEAQ-ITC analysis software (version 1.41, provided by the supplier) and fitted with the one-site model. In a control assay, *Bm*MDH2 was treated with 20 mM EDTA in glycine-NaOH buffer (100 mM, pH 9.5) for 2 h to chelate the metal ions in *Bm*MDH2. Subsequently, the buffer was exchanged to Tris-HCl (200 mM, pH 8.0) using the Amicon Ultra-15 spin filter, and the binding of ADPR to *Bm*MDH2 free of metal ion was analyzed using ITC as described above.

### Crystallization, data collection, and structure determination

The conditions for protein crystallization were screened through the sitting-drop vapor diffusion method at 18 °C using an automated protein crystallization workstation (Art Robbins Instruments, Gryphon LCP). The apo-*Bm*MDH2 crystals used in this study were obtained from a drop consisting of 2 μl protein solution (30 mg/ml) and 1 μl reservoir solution containing 0.2 M sodium acetate, 0.1 M Bis-Tris propane (pH 7.0), and 20% (w/v) PEG 3350. The crystals of *Bm*MDH2-ADPR complex were obtained from a drop consisting of 2 μl protein solution (30 mg/ml, 0.75 mM) containing 2 mM ADPR and 1 μl reservoir solution containing 0.2 M sodium formate, 0.1 M Bis-Tris propane (pH 6.0), and 15% (w/v) PEG 3350. The crystals of *Bm*MDH2-NAD^+^ complex were obtained from a drop consisting of 2 μl protein solution (30 mg/ml, 0.75 mM) containing 2 mM NAD^+^ and 1 μl reservoir solution containing 0.2 M sodium formate, 0.1 M Bis-Tris propane (pH 6.5), and 20% (w/v) PEG 3350. The crystals of apo-ACT were prepared by mixing the protein solution (90 mg/ml) in 20 mM Tris-HCl (pH 7.5) containing 150 mM NaCl with an equal volume of a reservoir solution. Two kinds of reservoir solutions were used for the crystallization of ACT: one contained 0.1 M MES monohydrate (pH 5.2) and 18% (w/v) polyethylene glycol 400, while the other comprised 0.1 M MMT buffer (pH 5.0) and 25% (w/v) PEG 1500.

Crystals were cryoprotected in the reservoir solution supplemented with 10% glycerol and, if necessary, 2 mM ADPR or NAD^+^. The crystals were then flash-frozen in liquid nitrogen and mounted prior to X-ray diffraction. X-ray diffraction data were collected at BL10U2 (for ACT, *Bm*MDH2-ADPR complex) and BL02U1 (for *Bm*MDH2 and *Bm*MDH2-NAD^+^ complex) beamlines of the Shanghai Synchrotron Radiation Facility. The collected data were indexed, integrated, and scaled using the X-ray Detector Software (XDS) package (47).

The structures were determined using molecular replacement *via* Phaser in the CCP4i program suite (48, 49). Atomic coordinates of nucleoside diphosphate sugar hydrolase (PDB ID: 5C7T) (30), and 1,3-propanediol dehydrogenase (PDB ID: 4FR2) (50) were used as the search models for ACT and *Bm*MDH2, respectively. The models were subjected to iterative rounds of rebuilding and refinement in Coot and Phenix (51, 52). For the *Bm*MDH2–NAD^+^ complex structure, the nicotinamide moiety of NAD^+^ exhibited relatively weak electron density, likely due to partial degradation of NAD^+^ to ADPR during crystallization. In two subunits of the *Bm*MDH2 decamer, the electron density corresponding to the nicotinamide group was missing, and ADPR was modeled instead in these chains. Data collection and refinement statistics were summarized in Table 1. The atomic coordinates and structure factors have been deposited in the Protein Data Bank: apo-ACT (PDB: 8WV3, 9JAX), apo-*Bm*MDH2 (PDB: 9JAW), *Bm*MDH2-ADPR complex (PDB: 8ZRL), *Bm*MDH2-NAD^+^ complex (PDB: 9JAV). All figures of the protein model were prepared with Pymol (www.pymol.org/).

**Table 1:** X-ray data collection and refinement statistics

Name	Apo-ACT	Apo-ACT2	Apo-*Bm*MDH2	*Bm*MDH2-ADPR complex	*Bm*MDH2-NAD^+^ complex
PDB ID	8WV3	9JAX	9JAW	8ZRL	9JAV
Data collection					
Wavelength (Å)	0.979183	0.979183	0.979183	0.979183	0.979183
Space group	*P*12_1_1	*P*12_1_1	*P*2_1_2_1_2_1_	*P*2_1_2_1_2_1_	*P*2_1_2_1_2_1_
Cell dimensions (Å)	a = 76.83 b = 96.88 c = 103.99	a = 59.71 b = 98.31 c = 73.50	a = 83.33 b = 203.53 c = 254.94	a = 83.57 b = 204.41 c = 258.14	a = 83.52 b = 204.10 c = 258.07
Resolution (Å)^a^	1.98 (2.08–1.98)	2.40 (2.49–2.40)	2.80 (2.85–2.80)	2.60 (2.64–2.60)	2.60 (2.64–2.60)
No of measured reflections	594,778	204,575	1,466,590	1,038,540	1,830,721
No of unique reflections^a^	102,810 (13,895)	31,100 (3256)	107,616 (5238)	136,650 (6650)	136,361 (6653)
Redundancy^a^	5.8 (5.9)	6.6 (6.5)	13.6 (14.1)	7.6 (6.9)	13.4 (13.7)
Completeness (%)^a^	97.5 (90.6)	99.7 (99.8)	100.0 (100.0)	100.0 (100.0)	100.0 (100.0)
Average (I/σ)^a^	11.3 (2.4)	15.7 (4.2)	12.6 (3.1)	14.3 (2.4)	13.8 (2.7)
R_merge_ (%)^a^^,^^b^	0.089 (0.833)	0.079 (0.663)	0.242 (1.199)	0.355 (0.629)	0.184 (1.202)
Refinement					
No of reflections	102,409 (10,388)	31,046 (3078)	107,482 (10,627)	136,469 (4258)	136,211 (13,527)
R_work_/R_free_^c^	0.1985/0.2440	0.1865/0.2392	0.2002/0.2462	0.1956/0.2404	0.1754/0.2397
Clashscore	6.05	7.12	8.65	8.33	8.41
No of non-H atoms					
Protein	10,997	5754	28,470	28,481	28,470
Waters	383	189	31	712	1133
Ligand	52	0	11	370	434
Average B factor [A^2^]	47	57.0	42.7	41.7	44.1
RMS deviations					
Bond lengths (Å)	0.0086	0.0083	0.0085	0.0080	0.0086
Bond angles (°)	0.99	0.99	1.38	1.42	1.02
Ramachandran plot favored (%)	96.03	96.81	96.86	97.33	94.92
Ramachandran plot allowed (%)	3.97	2.90	2.77	2.36	4.61
Ramachandran plot outliers (%)	0	0.29	0.37	0.31	0.47

a Numbers in parentheses are values for the highest-resolution shell.

b $R_{\text{merge}}=\sum_{\text{hkl}}\sum_{i}\left|I_{i}-<I>\left|/\sum_{\text{hkl}}\sum_{i}\right|<I>\right|$, where I_i_ is the intensity for the *i*^th^ measurement of an equivalent reflection with indices h, k, and l.

c *R*_free_ was calculated with the 5% of reflections set aside randomly throughout the refinement.

## Data availability

All structural data presented are publicly available. Crystal structures are deposited to the PDB with accession codes of 8WV3, 9JAX, 9JAW, 8ZRL, and 9JAV. All other data are available upon request.

## Supporting information

This article contains supporting information (30, 32).

## Conflict of interest

The authors declare that they have no conflicts of interest with the contents of this article.
