# Supplementary material for: ADP-ribose is a competitive inhibitor of methanol dehydrogenases from Bacillus methanolicus
Source: J Biol Chem. 2025 Aug 14;301(9):110599. doi: 10.1016/j.jbc.2025.110599 (PMC12926040; doi:10.1016/j.jbc.2025.110599)
Supplement: Supporting Information [file mmc1.docx]

Supporting Information

ADP-ribose is a competitive inhibitor of methanol dehydrogenases from *Bacillus methanolicus*

Bao-Di Ma, Jia-Yi Li, Jian-He Xu, Tao Yu and Xu-Dong Kong*

#

# SUPPLEMENTARY RESULTS

## The metal-dependence of *Bm*MDH2

As a first step, different metal ions were directly incubated with *Bm*MDH2 to determine their impact on its activity. The purified *Bm*MDH2 was diluted to 10 μM in glycine-NaOH buffer (100 mM, pH 9.5) containing 1 mM of various metal ions. After incubation on ice for 1 h, the enzymatic activities were measured in glycine-NaOH buffer (100 mM, pH 9.5) containing 3 mM NAD^+^, 1 μM *Bm*MDH2, 0.1 mM various metal ions and 50 mM propanol at 37ºC. As shown in Supplementary Fig. 12a, Zn^2+^ or Cu^2+^ significantly inhibited the activity of *Bm*MDH2, suggesting that metal ions at the active site of *Bm*MDH2 have been replaced by catalytically nonfunctional metal ions (Zn^2+^ and Cu^2+^). The presence of Mn^2+^ or Co^2+^ significantly enhanced the activity of *Bm*MDH2, suggesting that either the amount of native metal ion in purified *Bm*MDH2 was insufficient or the native metal ion was not preferred for catalysis. The incubation with Fe^2+^ exhibited negligible effect on *Bm*MDH2 activity, while metal ions Ni^2+^, Ca^2+^ or Mg^2+^ caused slightly decreased *Bm*MDH2 activity. The impact of these metal ions on the activity of *Bm*MDH2 can be attributed to both the binding affinity of these metal ions for *Bm*MDH2 and the activity of the complex formed between *Bm*MDH2 and the metal ions.

To further characterize the specific activity of *Bm*MDH2 containing different metal ions, the purified *Bm*MDH2 was treated with EDTA to remove the metal ions it coordinated during expression or purification. The protein was incubated with 10 equivalents of EDTA in glycine-NaOH buffer (100 mM, pH 9.5) on the ice for 1 hour. Subsequently, the EDTA was removed from the protein using the HiTrap desalting column (Cytiva, CAT#17-1408-01). Following this incubation, the activity of *Bm*MDH2 was completely lost, indicating that metal ions are essential for its dehydrogenase activity (Supplementary Fig. 12b). The *Bm*MDH2 free of metal ions at 10 μM was incubated in glycine-NaOH buffer (100 mM, pH 9.5) containing 1 mM of various metal ions on ice for 1 h, followed by the analysis of the activity. Some of these metal ions resulted in the recovery of activity, and Mn^2+^ leads to the highest activity of *Bm*MDH2 (1.04 ± 0.02 U/mg). In contrast, Zn^2+^, Cu^2+^, Mg^2+^ or Ca^2+^ didn’t restore the activity of *Bm*MDH2. Overall, the above results indicated the optimum metal ion for *Bm*MDH2 activity was Mn^2+^, which was also supplied in the rest of the research.

## The stability of NADx activation effect at different pH

As described in the main text, we observed that the activation effect of filtered ACT/NAD^+^ mixtures (NADx) was unstable after incubation at r.t. In order to understand the mechanism behind it, we characterized the stability of NADx in different buffers. The activation effect of purified NADx was examined after 30 minutes of incubation in various buffers including water, Tris-HCl buffer (pH 8.0 and pH 8.8) and glycine-NaOH buffer (pH 9.5). As shown in Supplementary Fig. 13, the activity of *Bm*MDH2 in all samples decreased after the incubation when compared with the fully activated *Bm*MDH2 in the corresponding buffer containing ACT. After incubation of NADx in glycine-NaOH buffer (pH 9.5) for 30 minutes, the activity of *Bm*MDH2 significantly decreased to merely 8.4% as the fully activated form. In contrast, the NADx in water or Tris-HCl buffer (pH 8.0) exhibited a higher activation efficiency, which resulted in 80% and 55% of activity compared to the fully activated *Bm*MDH2. The results of stability characterization presented a trend that higher pH accelerated the loss of the activation effect of NADx.

## Key residues of ACT for ADPR hydrolysis

The potential general base or the residues responsible for the binding of metal ions and water around the active sites of ACT were identified according to the structures and mechanisms of Nudix hydrolases, and mutated by site-specific mutagenesis^1,2^ (Supplementary Table 1). Specifically, arginine and glutamic acid were mutated to alanine and glutamine, respectively. In order to evaluate the effects of these residues, the mutants were purified and their specific activities toward ADPR hydrolysis were analyzed (Supplementary Fig. 14 and Table 2). Among the 5 mutants characterized, ACT_E93Q_ and ACT_E97Q_ completely lost the ability to hydrolyze ADPR, and ACT_R92A_ exhibited an activity of 2.9 U/mg, corresponding to 4.7% of the activity of wild-type ACT. These results suggested that E93, E97, and R92 are crucial for ACT to bind and/or catalyze the hydrolysis of ADPR.

# SUPPLEMENTARY MATERIALS AND METHODS

**Sequences of the genes and proteins**

**ORF sequence of ACT**: ATGGGCAAACTGTTTGAAGAGAAAACCATTAAAACCG AGCAGATTTTTAGCGGTCGTGTTGTTAAACTGCAGGTTGATGATGTTGAACTGCCGAATGGTCAGACCAGCAAACGTGAAATTGTTCGTCATCCGGGTGCAGTTGCAGTTATTGCAATTACCAACGAAAACAAGATCGTGATGGTTGAGCAGTATCGTAAACCGCTGGAAAAATCCATTGTTGAAATTCCGGCAGGCAAACTGGAAAAAGGTGAAGATCCGCGTATTACCGCACTGCGTGAACTGGAAGAAGAAACCGGTTATGAATGTGAGCAGATGGAATGGCTGATTAGCTTTGCAACCAGTCCGGGTTTTGCAGATGAAATCATTCATATCTATGTGGCCAAAGGCCTGAGCAAGAAAGAAAATGCAGCAGGTCTGGATGAAGATGAATTTGTGGATCTGATTGAACTGACCCTGGATGAGGCACTGCAGTATATCAAAGAACAGCGTATCTATGATAGCAAAACCGTTATTGCAGTTCAGTACTTACAGCTGCAAGAAGCCCTGAAAAACAAACTCGAGCACCACCACCACCACCACTGA

**Amino acid sequence of ACT**: MGKLFEEKTIKTEQIFSGRVVKLQVDDVELPNGQ TSKREIVRHPGAVAVIAITNENKIVMVEQYRKPLEKSIVEIPAGKLEKGEDPRITALRELEEETGYECEQMEWLISFATSPGFADEIIHIYVAKGLSKKENAAGLDEDEFVDLIELTLDEALQYIKEQRIYDSKTVIAVQYLQLQEALKNKLEHHHHHH*

**ORF sequence of *Bm*MDH1**: ATGACCACCAACTTTTTTATTCCGCCGGCGAGCG TGATTGGCCGCGGCGCGGTGAAAGAAGTGGGCACCCGCCTGAAACAGATTGGCGCGAAAAAAGCGCTGATTGTGACCGATGCCTTTCTGCACAGCACCGGCCTGAGCGAAGAAGTGGCGAAAAACATTCGCGAAGCGGGCGTGGACGTGGCGATTTTTCCGAAAGCGCAGCCGGATCCGGCGGATACCCAAGTGCATGAAGGCGTGGATGTGTTTAAACAAGAAAACTGCGATAGCCTGGTGAGCATTGGCGGTGGCAGCAGCCATGATACCGCGAAAGCGATTGGCCTGGTGGCGGCGAACGGCGGCCGCATTAACGATTATCAAGGCGTGAACAGCGTGGAAAAACCGGTGGTGCCGGTGGTGGCGATTACCACGACCGCGGGCACCGGCAGCGAAACCACGAGCCTGGCGGTGATTACCGATAGCGCGCGCAAAGTGAAAATGCCGGTGATTGATGAAAAAATTACCCCGACCGTGGCGATTGTGGATCCGGAACTGATGGTGAAAAAACCGGCGGGCCTGACCATTGCGACCGGCATGGATGCGCTGAGCCATGCGATTGAAGCGTATGTGGCGAAAGGCGCGACCCCGGTGACCGACGCCTTTGCGATTCAAGCGATGAAACTGATTAACGAATATCTGCCGAAAGCGGTGGCGAACGGCGAAGATATTGAAGCCCGTGAAAAAATGGCGTATGCGCAGTATATGGCGGGCGTGGCGTTTAACAACGGCGGCCTGGGCCTGGTGCATAGCATTAGCCATCAAGTGGGCGGCGTGTATAAACTGCAGCATGGCATTTGCAACAGCGTGAACATGCCGCATGTGTGCGCGTTTAACCTGATTGCGAAAACCGAACGCTTTGCGCATATTGCCGAATTACTGGGCGAGAACGTGGCGGGCCTGAGTACCGCGGCCGCGGCGGAACGCGCGATTGTGGCGCTGGAACGCATTAACAAAAGCTTTGGCATTCCGAGCGGCTATGCGGAAATGGGCGTGAAAGAAGAAGATATTGAGCTGTTAGCGAAAAACGCGTATGAAGATGTGTGCACGCAGAGCAACCCGCGCGTGCCGACCGTGCAAGATATTGCGCAGATTATTAAAAACGCGATGCTCGAGCACCACCACCACCACCACTGA

**Amino acid sequence of *Bm*MDH1**: MTTNFFIPPASVIGRGAVKEVGTRLKQIGAKKA LIVTDAFLHSTGLSEEVAKNIREAGVDVAIFPKAQPDPADTQVHEGVDVFKQENCDSLVSIGGGSSHDTAKAIGLVAANGGRINDYQGVNSVEKPVVPVVAITTTAGTGSETTSLAVITDSARKVKMPVIDEKITPTVAIVDPELMVKKPAGLTIATGMDALSHAIEAYVAKGATPVTDAFAIQAMKLINEYLPKAVANGEDIEAREKMAYAQYMAGVAFNNGGLGLVHSISHQVGGVYKLQHGICNSVNMPHVCAFNLIAKTERFAHIAELLGENVAGLSTAAAAERAIVALERINKSFGIPSGYAEMGVKEEDIELLAKNAYEDVCTQSNPRVPTVQDIAQIIKNAMLEHHHHHH*

**ORF sequence of *Bm*MDH2**: ATGACCAATACACAGAGCGCATTTTTCATGCCGA GCGTTAACCTGTTTGGTGCAGGTAGCGTTAATGAAGTTGGCACCCGTCTGGCAGATCTGGGTGTTAAAAAGGCACTGCTGGTTACCGATGCAGGTCTGCATGGTCTGGGTCTGAGCGAGAAAATTAGCAGCATTATTCGTGCAGCCGGTGTTGAAGTTAGCATTTTTCCGAAAGCAGAACCGAATCCGACCGATAAAAATGTTGCAGAAGGTCTGGAAGCATATAATGCCGAAAATTGCGATAGCATTGTTACCTTAGGTGGTGGTAGCAGCCATGATGCAGGTAAAGCAATTGCACTGGTTGCAGCAAATGGTGGCAAAATCCATGATTATGAAGGTGTGGATGTTAGCAAAGAACCGATGGTTCCGCTGATTGCAATTAATACCACCGCAGGCACCGGTAGCGAACTGACCAAATTTACCATTATTACCGATACCGAGCGCAAAGTGAAAATGGCGATTGTTGATAAACATGTTACCCCGACACTGAGCATTAATGATCCGGAACTGATGGTTGGTATGCCTCCGAGCCTGACCGCAGCAACCGGTCTGGATGCACTGACCCATGCAATTGAAGCCTATGTGAGTACCGGTGCAACCCCGATTACAGATGCACTGGCAATTCAGGCCATTAAAATCATCAGCAAATATCTGCCTCGTGCAGTTGCAAATGGCAAAGATATTGAAGCACGTGAGCAGATGGCATTTGCACAGAGCCTGGCAGGTATGGCATTCAATAATGCCGGTCTGGGTTATGTTCATGCCATTGCACATCAGTTAGGTGGCTTTTATAACTTTCCGCATGGTGTTTGTAATGCAGTTCTGCTGCCGTATGTTTGTCGCTTTAATCTGATTAGCAAAGTGGAACGCTATGCAGAAATTGCAGCCTTTCTGGGTGAAAATGTTGATGGTCTGAGCACCTATGATGCAGCAGAAAAAGCAATTAAAGCCATTGAACGTATGGCCAAAGATCTGAATATTCCGAAAGGCTTTAAAGAGCTGGGAGCCAAAGAAGAAGATATCGAAACCCTGGCCAAAAACGCAATGAAAGATGCATGTGCCCTGACCAATCCGCGTAAACCGAAACTGGAAGAAGTTATCCAGATTATCAAAAATGCCATGCTCGAGCACCACCACCACCACCACTGA

**Amino acid sequence of *Bm*MDH2**: MTNTQSAFFMPSVNLFGAGSVNEVGTRLADLG VKKALLVTDAGLHGLGLSEKISSIIRAAGVEVSIFPKAEPNPTDKNVAEGLEAYNAENCDSIVTLGGGSSHDAGKAIALVAANGGKIHDYEGVDVSKEPMVPLIAINTTAGTGSELTKFTIITDTERKVKMAIVDKHVTPTLSINDPELMVGMPPSLTAATGLDALTHAIEAYVSTGATPITDALAIQAIKIISKYLPRAVANGKDIEAREQMAFAQSLAGMAFNNAGLGYVHAIAHQLGGFYNFPHGVCNAVLLPYVCRFNLISKVERYAEIAAFLGENVDGLSTYDAAEKAIKAIERMAKDLNIPKGFKELGAKEEDIETLAKNAMKDACALTNPRKPKLEEVIQIIKNAMLEHHHHHH*

**ORF sequence of *Cn*MDH2 CT4-1**: ATGACACATTTAAATATTGCTAATAGAGTAGAT

TCATTCTTCATCCCATGTGTAACATTATTTGGTCCTGGTTGTGTAAGAGAAACAGGTGTAAGAGCTAGATCATTAGGTGCTAGAAAGGCGTTGATTGTAACAGATGCTGGTTTACATAAGATGGGCTTATCAGAAGTAGTAGCTGGACACATAAGGGAGGCAGGTTTACAAGCTGTAATATTCCCAGGCGCGGAGCCCAACCCGACCGACGTCAACGTTCACGACGGTGTCAAGTTATTTGAGCGGGAAGAATGTGATTTCATCGTTTCATTAGGTGGTGGTTCATCACATGATTGTGCTAAAGGTATTGGTTTAGTAACAGCTGGTGGTGGCCACATCAGGGACTACGAAGGTATTGATAAATCAACAGTACCTATGACGCCGTTAATAAGTATCAATACAACAGCTGGCACGGCCGCGGAGATGACCCGTTTCTGCATAATAACCAACTCGTCCAACCACGTAAAGATGGTCATTGTAGATTGGAGATGTACTCCGCTAATAGCAATCGATGATCCTTCATTAATGGTAGCTATGCCTCCTGCTTTAACGGCCGCGACCGGCATGGATGCTTTAACACATGCTATTGAAGCTTATGTATCAACCGCCGCAACTCCAATCACCGATGCTTGTGCTGAGAAGGCAATAGTATTAATTGCTGAATGGTTACCTAAAGCTGTAGCTAATGGTGATTCAATGGAAGCTAGAGCTGCTATGTGTTATGCTCAATATTTAGCTGGTATGGCTTTCAACAACGCTTCATTAGGTTATGTACATGCTATGGCTCATCAATTAGGTGGTTTCTACAACTTACCTCATGGTGTATGTAATGCTATCTTGCTACCTCATGTATCAGAATTTAATTTAATTGCTGCTCCTGAAAGATATGCTAGAATTGCTGAATTATTAGGTGAGAACATAGGTGGTTTATCAGCTCATGATGCTGCTAAAGCTGCTGTATCAGCTATTAGAACATTATCAACATCAATTGGTATTCCTGCTGGTTTAGCTGGTTTAGGTGTAAAGGCAGACGATCATGAAGTAATGGCTTCAAATGCTCAGAAGGACGCCTGTATGTTAACAAATCCTAGAAAGGCCACGTTAGCTCAAGTAATGGCTATATTCGCGGCAGCTATGTGA

**Amino acid sequence of *Cn*MDH2 CT4-1**: MGMTHLNIANRVDSFFIPCVTLFGPGCV RETGVRARSLGARKALIVTDAGLHKMGLSEVVAGHIREAGLQAVIFPGAEPNPTDVNVHDGVKLFEREECDFIVSLGGGSSHDCAKGIGLVTAGGGHIRDYEGIDKSTVPMTPLISINTTAGTAAEMTRFCIITNSSNHVKMVIVDWRCTPLIAIDDPSLMVAMPPALTAATGMDALTHAIEAYVSTAATPITDACAEKAIVLIAEWLPKAVANGDSMEARAAMCYAQYLAGMAFNNASLGYVHAMAHQLGGFYNLPHGVCNAILLPHVSEFNLIAAPERYARIAELLGENIGGLSAHDAAKAAVSAIRTLSTSIGIPAGLAGLGVKADDHEVMASNAQKDACMLTNPRKATLAQVMAIFAAAMLEHHHHHH*

# Supplementary Tables

Table S1. Primers used in this study

| Enzyme | Primer sequences | |
| --- | --- | --- |
| *Cn*MDH CT4-1 | F^a^ | 5’-catgccatgggcatgacacatttaaatattgctaatag-3’ |
|  | R | 5’-ccctcgagcatagctgccgcgaatatagccattac-3’ |
| *Bm*MDH2_S101G_ | F | 5’-ttaccttaggtggtggtggcagccatgatgcag-3’ |
|  | R | 5’-ctgcatcatggctgccaccaccacctaaggtaa-3’ |
| ACT_R92A_ | F | 5’-ccgcgtattaccgcactggctgaactggaagaagaaac-3’ |
|  | R | 5’-gtttcttcttccagttcagccagtgcggtaatacgcgg-3’ |
| ACT_E93Q_ | F | 5’-gtattaccgcactgcgtcagctggaagaagaaaccgg-3’ |
|  | R | 5’-ccggtttcttcttccagctgacgcagtgcggtaatac-3’ |
| ACT_E96Q_ | F | 5’-cgcactgcgtgaactggaacaggaaaccggttatgaatgtg-3’ |
|  | R | 5’-cacattcataaccggtttcctgttccagttcacgcagtgcg-3’ |
| ACT_E97Q_ | F | 5’-ccgcactgcgtgaactggaagaacagaccggttatgaa-3’ |
|  | R | 5’-ttcataaccggtctgttcttccagttcacgcagtgcgg-3’ |
| ACT_E143Q_ | F | 5’-atgaagatcagtttgtggatctgattg-3’ |
|  | R | 5’-tccacaaactgatcttcatccagacc-3’ |

^a^F and R indicates the forward and reverse primers used in the PCR reactions for cloning or mutagenesis.

^b^The mutagenic nucleotides were highlighted in red.

Table S2. The specific activity of ACT mutants for the hydrolysis of ADPR^a^

| Entry | Mutant | Specific activity  (U/mg) |
| --- | --- | --- |
| 1 | WT | 61.2 ± 0.5 |
| 2 | R92A | 2.9 ± 0.7 |
| 3 | E93Q | n.d. ^b^ |
| 4 | E96Q | 36 ± 5 |
| 5 | E97Q | n.d. |
| 7 | E143Q | 10 ± 2 |

^a^The specific activities of purified ACT and its mutants were assessed in a reaction mixture containing 100 μL of 100 mM Tris-HCl buffer (pH 8.0), 5 mM MgCl_2_, 2 nM ACT, and 400 μM ADPR. After a 5-minute incubation at 25°C, the reaction was terminated by the addition of 10 μL of 3 M H₂SO₄. The products were subsequently analyzed by HPLC.

^b^n.d. not detected.

**Supplementary Figures**


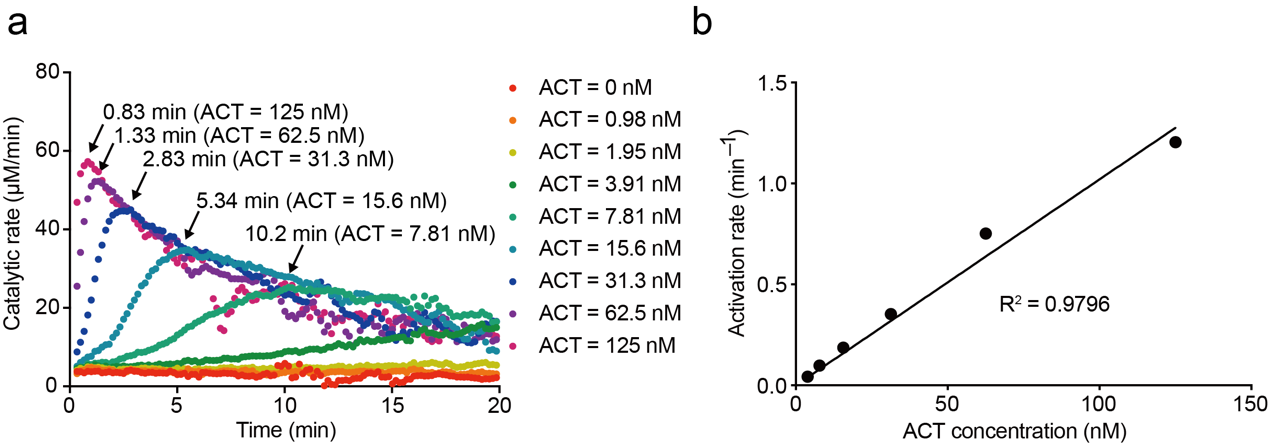


Fig. S1 The effect of ACT concentration on the activation rate of *Bm*MDH2**. a**, The effect of ACT concentration on the catalytic rate of *Bm*MDH2. The catalytic rate was calculated from the data shown in **Fig. 1*A***. The activation time required to reach the highest catalytic rate at each ACT concentration was labeled on the curve. **b**, The effect of ACT concentration on the activation rate of *Bm*MDH2. The activation rate was calculated as the reciprocal of activation time. ACT concentration and activation rate were used in regression analysis, resulting in a *R* squared value of 0.9796.


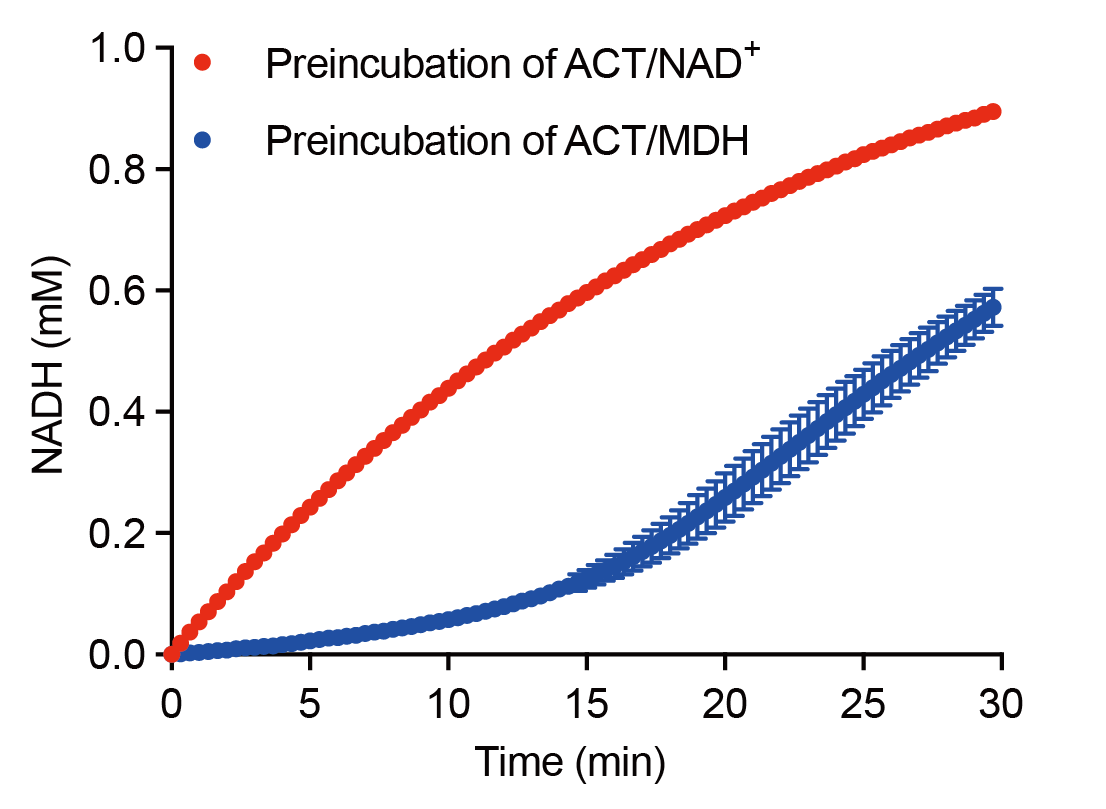


Fig. S2 Activation effects of pre-incubated ACT/NAD^+^ or ACT/*Bm*MDH2**.** ACT was pre-incubated with the NAD^+^ or *Bm*MDH2, respectively, in glycine-NaOH buffer (100 mM, pH 9.5) containing 5 mM MgCl_2_ at 25°C. After 30 minutes of incubation, the *Bm*MDH2 or NAD^+^ were added into the samples, to reach the same final reaction system containing 10 nM ACT, 5 mM MgCl_2_, 3 mM NAD^+^, 200 nM *Bm*MDH2, 0.5 mM MnCl_2_, and 20 mM ethanol. Data are means ± SDs of three measurements.

**
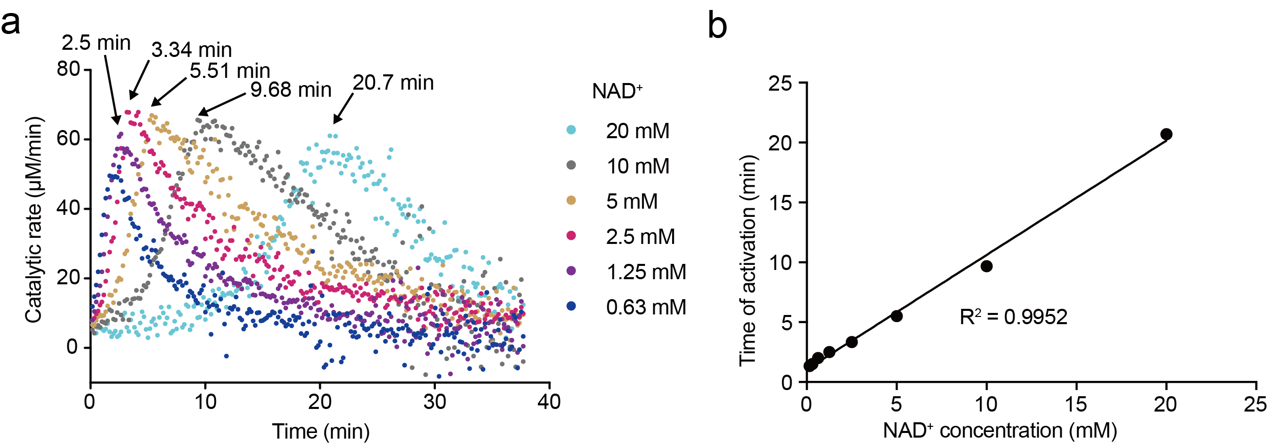
**

Fig. S3 The effect of NAD^+^ concentration on the activation rate of *Bm*MDH2**. a**, The effect of NAD^+^ concentration on the catalytic rate of *Bm*MDH2 in present of 50 nM ACT. The catalytic rate was calculated from the data shown in **Fig. 1*C***. The activation time required to reach the highest catalytic rate at each NAD^+^ concentration was labeled on the curve. **b**, The effect of NAD^+^ concentration on the activation time of *Bm*MDH2. NAD^+^ concentration and activation time were used in regression analysis, resulting in a *R* squared value of 0.9952.


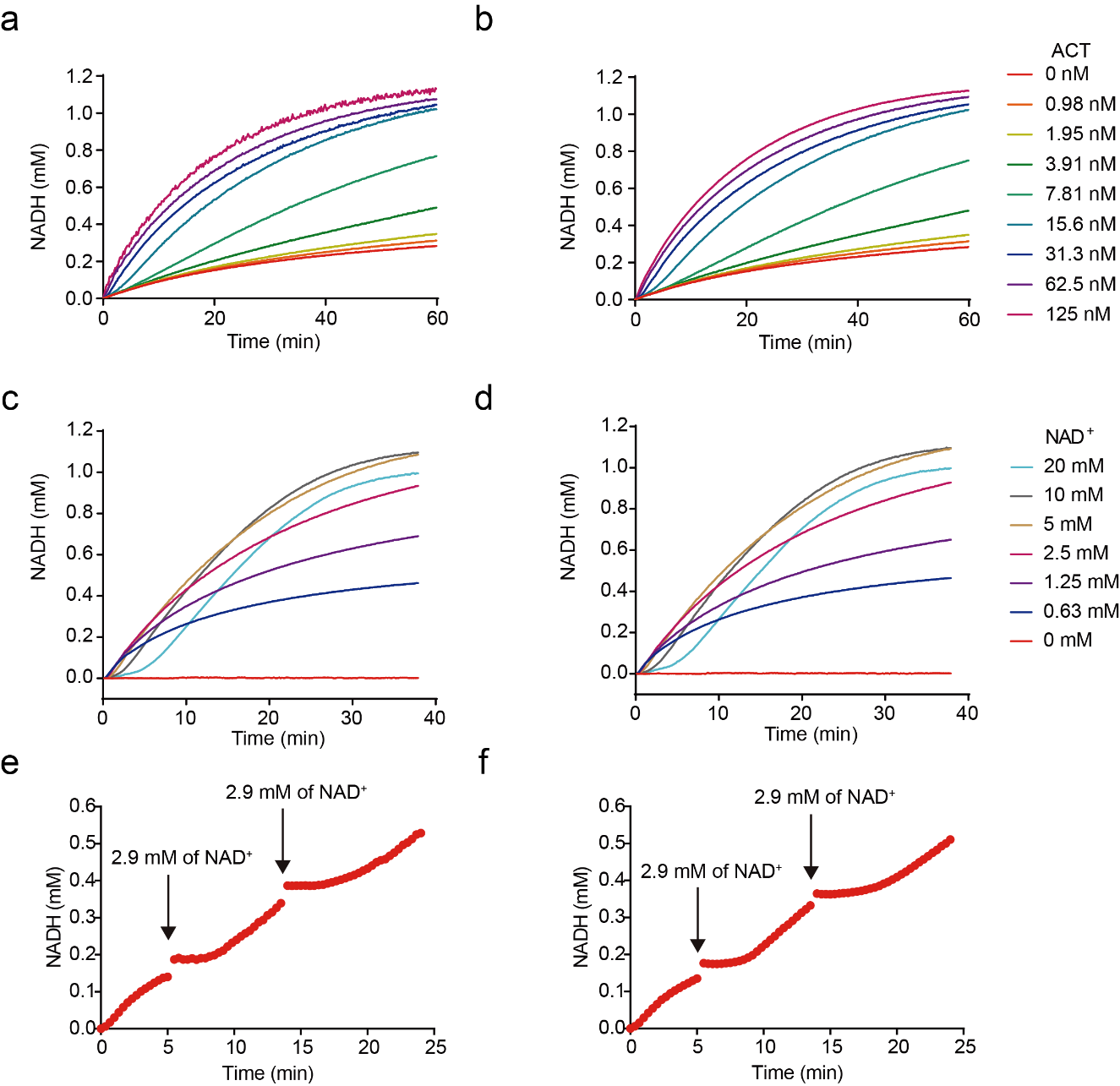


Fig. S4 **Effect of ACT and NAD^+^ concentration on *Bm*MDH2 activation.** **a-b**, Effect of ACT concentration on *Bm*MDH2 activation, corresponding to two independent replicates of the experiments shown in **Fig. 1*A***. **c-d**, Effect of NAD^+^ concentration on *Bm*MDH2 activation, corresponding to two independent replicates of the experiments shown in **Fig. 1*C***. **e-f**, Time-course of *Bm*MDH2 activation, corresponding to two independent replicates of the experiments shown in **Fig. 1*D***. All experiments in **a–f** were performed using NAD^+^ from a different commercial supplier. The overall activation trends are consistent with those in **Fig. 1**, although variations in activation kinetics are observed, likely due to differences in NAD^+^ purity.


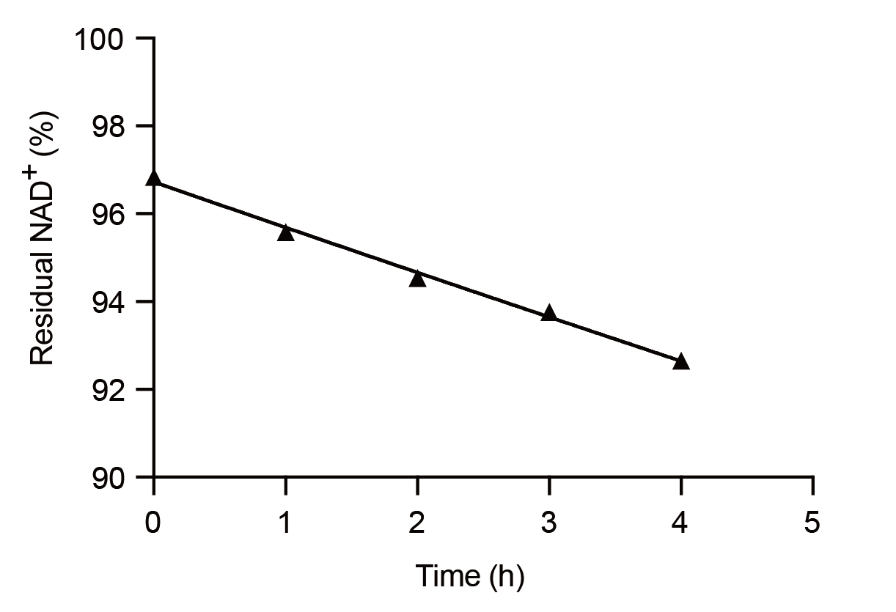


Fig. S5 The hydrolysis rate of NAD^+^ at 25ºC**.** NAD^+^ was dissolved in glycine-NaOH buffer (pH 9.5, 100 mM) at a concentration of 3 mM and incubated at 25ºC. Samples were withdrawn at various time intervals and analyzed by HPLC. The residual NAD^+^ was quantified according to the standard curves of NAD^+^. The data of residual NAD^+^ versus time were used in the non-linear fitting to the equation of one phase decay: Y = Y_0_ × (1 − e^−^*^k^*^∙t^), resulting in the hydrolysis rate constant *k* = 0.011 h^−1^ and a half-life of 64 h.

**
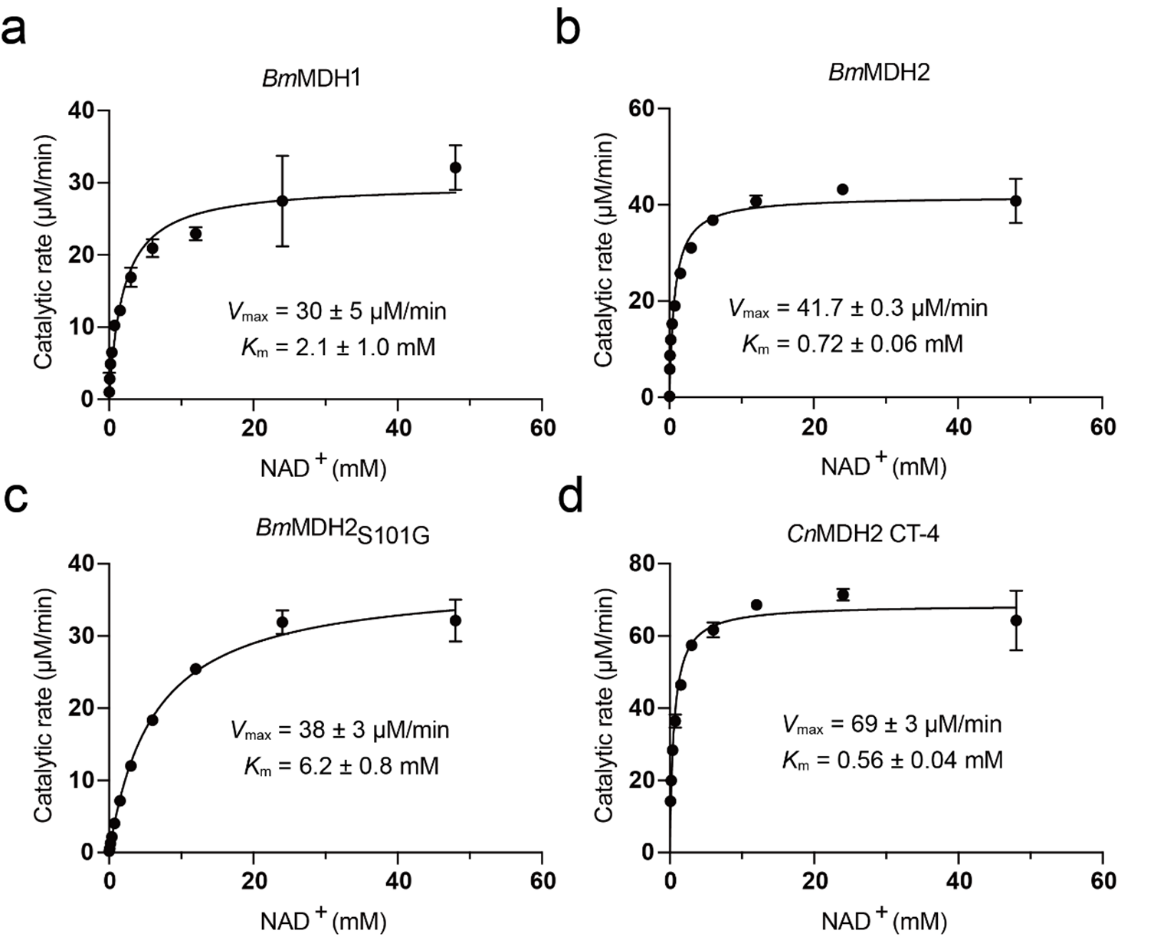
**

Fig. S6 Kinetics of *Bm*MDH1 (a), *Bm*MDH2 (b), *Bm*MDH2_S101G_ (c) and *Cn*MDH CT-4 (d) in the absence of ADPR**.** The kinetic assays were conducted in 200 μL Tris-HCl buffer (100 mM, pH 8.0) at 25ºC. The reaction mixture contained 40 mM ethanol, 0.5 mM MnCl_2_, 10 nM ACT, 5 mM MgCl_2_, adequate MDHs (*Bm*MDH1: 500 nM; *Bm*MDH2: 100 nM; *Bm*MDH2_S101G_: 100 nM; *Cn*MDH2 CT4: 100 nM), and 0 to 48 mM of NAD^+^. The ACT and MgCl_2_ were included to avoid the accumulation of ADPR in the reaction mixture. Data are means ± SDs of three measurements.

**
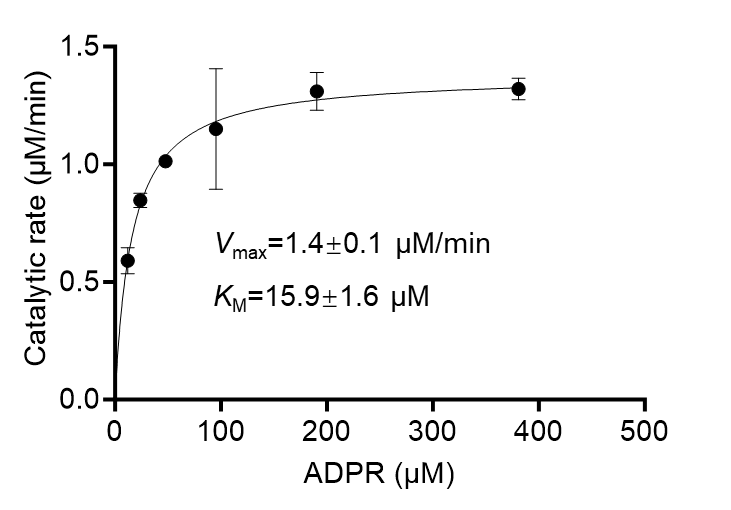
**

Fig. S7 The determination of kinetic parameters of ACT**.** The kinetic assays were performed in 100 μL Tris-HCl buffer (100 mM, pH 8.0) containing 5 mM MgCl_2_, 0.5 nM ACT and various concentrations of ADPR (ranging from 0 to 400 μM). Reactions were terminated by adding 10 μL of 3 M H_2_SO_4_ after incubation at 25^o^C for 5 minutes. Data are means ± SDs of three measurements.


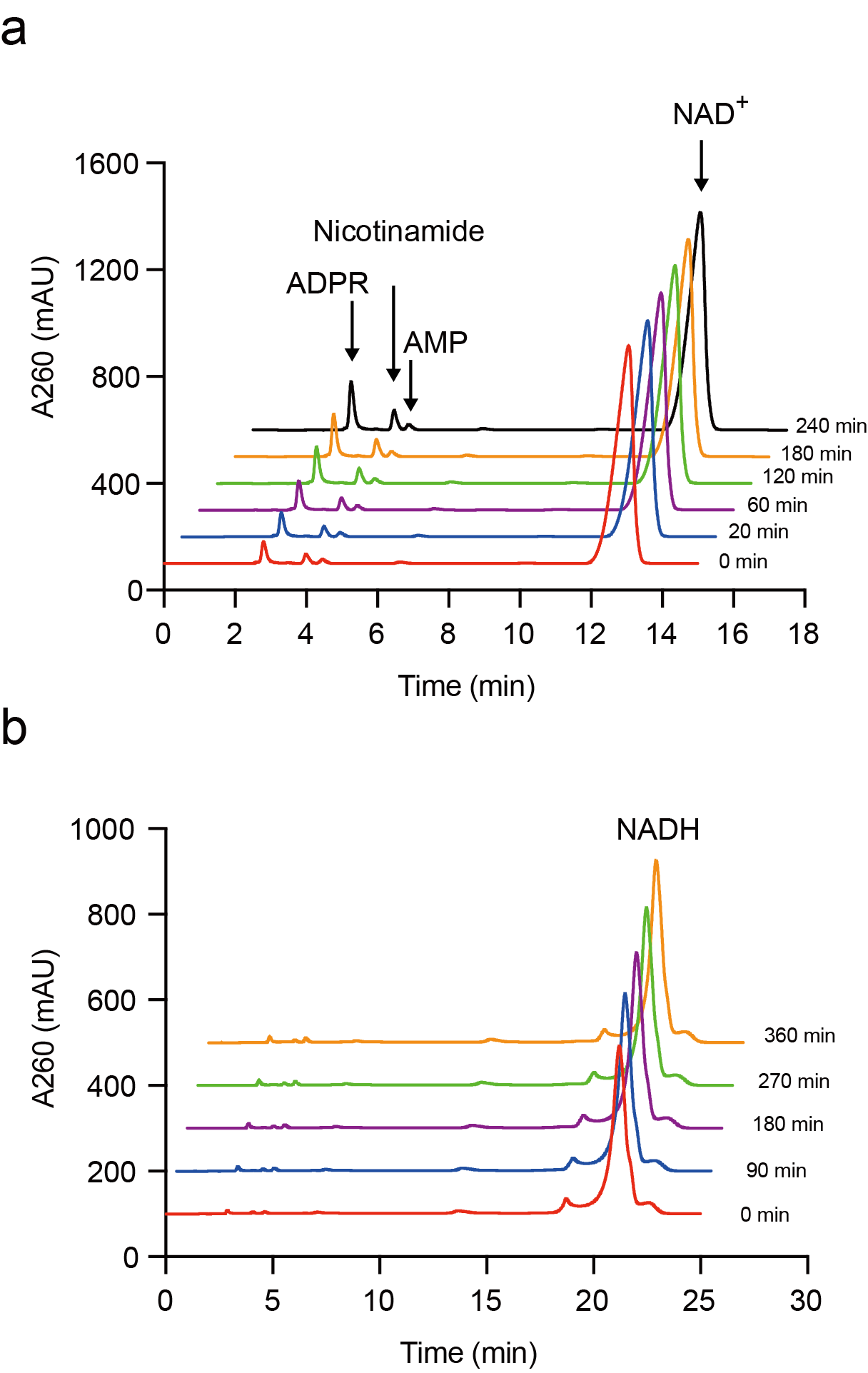


Fig. S8 Degradation progress of NAD^+^ (a) and NADH (b)**.** The commercial NAD^+^ (Sigma CAT#N7004) or NADH (Aladin CAT#N106933) were dissolved in glycine-NaOH buffer (100 mM, pH 9.5) and incubated at 25ºC. The samples were withdrawn and analyzed by HPLC at different time intervals.

**
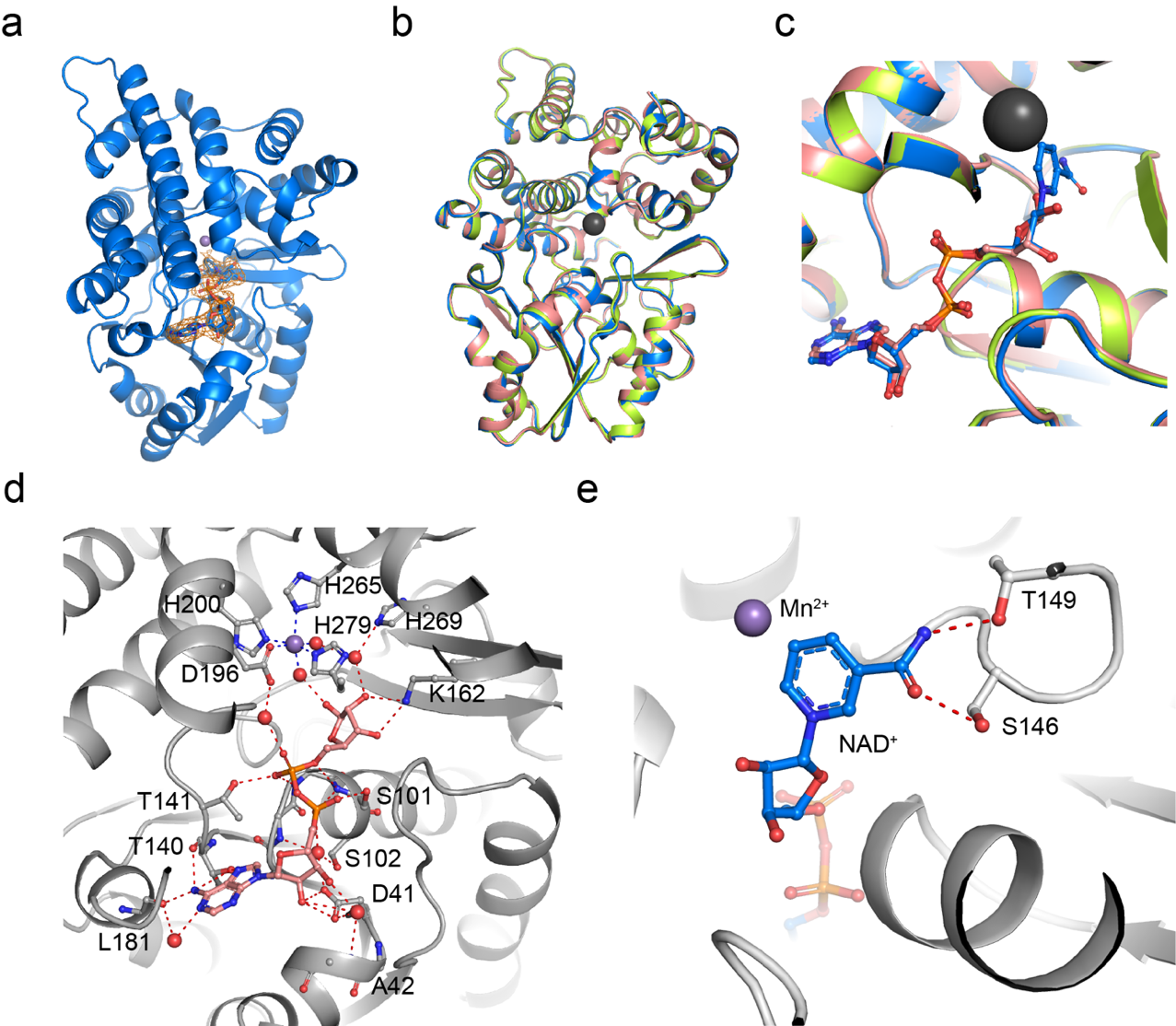
**

Fig. S9 Analysis of the binding modes of ADPR and NAD^+^**. a,** NAD^+^ and Mn^2+^ binding sites in the *Bm*MDH2 monomer. NAD^+^ and Mn^2+^ are shown as sticks and spheres, respectively. Omit map of NAD^+^ is contoured at 𝜎 = 1.0. **b**, Superposition of apo-*Bm*MDH2 (limon), *Bm*MDH2-ADPR (salmon) and *Bm*MDH2-NAD^+^ (marine) structures. RMSDs between *Bm*MDH2-NAD^+^/apo-*Bm*MDH2, *Bm*MDH2-NAD^+^/*Bm*MDH2-ADPR and *Bm*MDH2-ADPR/apo-*Bm*MDH2 pairs are 0.248, 0.248 and 0.286 Å, respectively. The Mn^2+^ at the active center of *Bm*MDH2 is shown as sphere in gray. **c,** Binding conformation of NAD^+^ and ADPR in the superposed *Bm*MDH2-NAD^+^ (marine) and *Bm*MDH2-ADPR (salmon) complex structures. The NAD^+^ and ADPR are shown as ball-and-stick model. The Mn^2+^ is shown as sphere. **d,** Hydrogen bonds between ADPR and *Bm*MDH2 in the complex structure, represented as red dashed lines. The Mn^2+^ and water molecules are shown as purple and red spheres, respectively. The coordinate bonds between Mn^2+^ and amino acids or water molecules are shown as blue dashed lines. ADPR and amino acids involved in the interactions are shown as ball-and-stick model. **e,** Hydrogen bonds between NAD^+^ and *Bm*MDH2 in the complex structure, represented as red dashed lines. Mn^2+^ is shown as sphere. NAD^+^ and amino acids involved in the interactions are shown as ball-and-stick model**.**


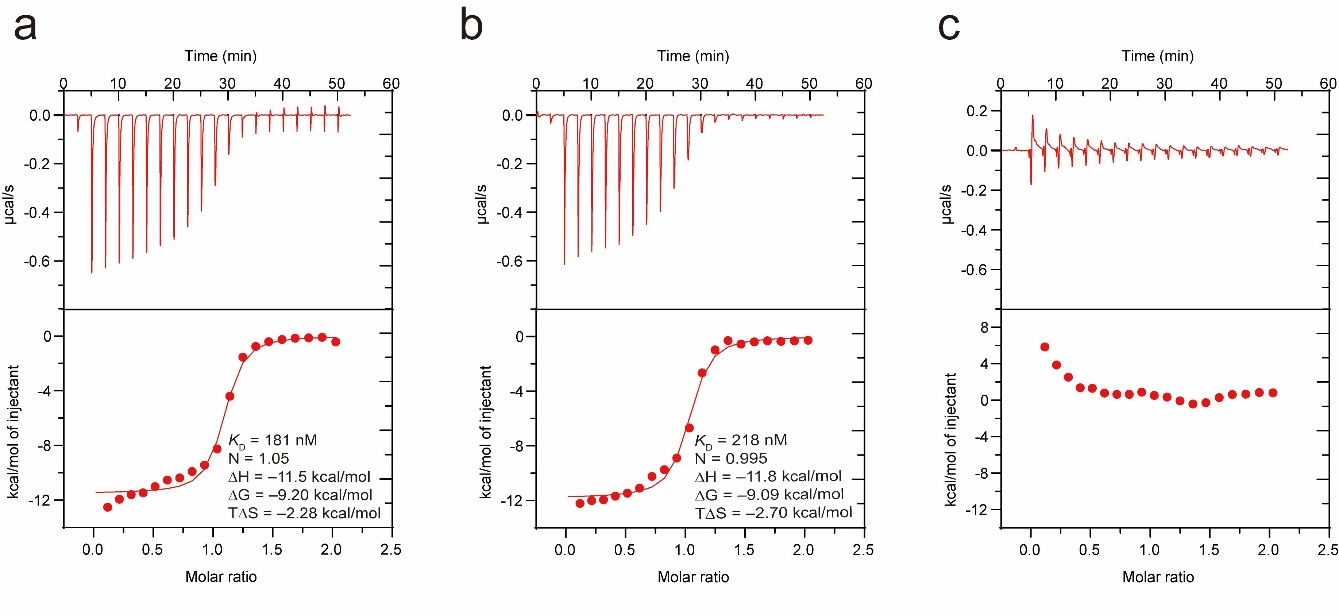


Fig. S10 ITC analysis of ADPR binding to *Bm*MDH2 (a, b) and EDTA-treated *Bm*MDH2 (c). a and b, ITC measurements were performed at 25°C using 200 μL of 30 µM *Bm*MDH2 in 200 mM Tris-HCl (pH 8.0) containing 0.5 mM MnCl_2_. A 300 µM ADPR solution in the same buffer was titrated into the sample at 0.5 µL/s with 150 s intervals, starting with an initial injection of 0.4 μL followed by 19 injections of 2 μL. Data were analyzed using MicroCal PEAQ-ITC analysis software and fitted with the one-site model. c, *Bm*MDH2 was treated with 20 mM EDTA in 100 mM glycine-NaOH buffer (pH 9.5) for 2 hours. Subsequently, the buffer was exchanged to 200 mM Tris-HCl (pH 8.0), and the binding of ADPR to metal-free *Bm*MDH2 was analyzed as described in (**a)** and (**b)**.

Fig. S11 The inhibition effect of ADPR on type I (*Sc*ADH) and type II (IPADH_M4_, *Cb*FDH, *Bm*GDH, *Ba*AlaDH) alcohol dehydrogenases**.**  NAD^+^ was used as the coenzyme in all assays at a final concentration of 0.5 mM. ACT was added at a final concentration of 1 μM to investigate its activation effect on various dehydrogenases. The ADPR was added at concentrations of 0.5 or 3 mM to evaluate the inhibitory effect of ADPR to the activity of different dehydrogenases. Data are means ± SDs of three measurements.


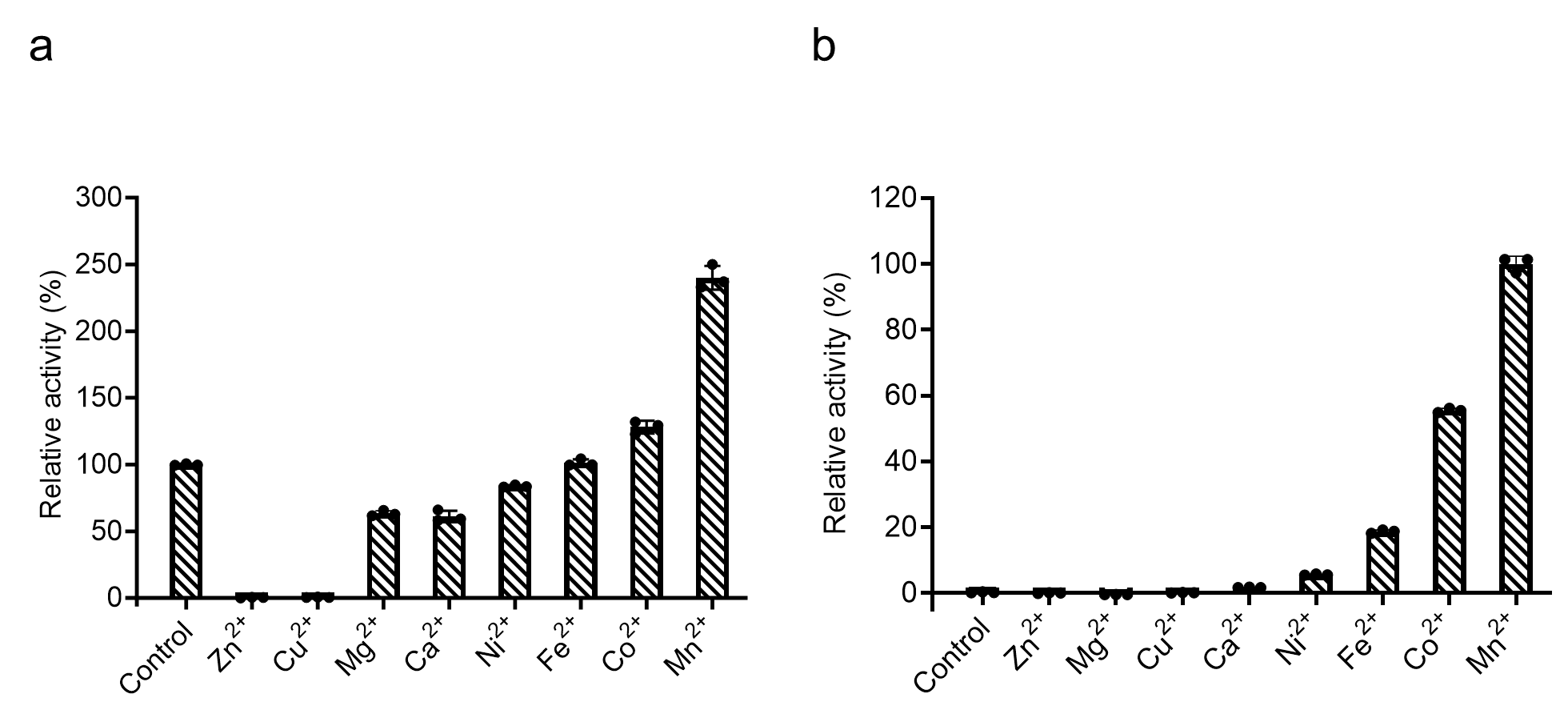


Fig. S12 The effect of metal ions on the activity of *Bm*MDH2**. a**, The effect of metal ions on the activity of *Bm*MDH2. The purified *Bm*MDH2 at 10 μM was incubated with 1 mM of various metal ions on ice for 1 h, the enzyme activities were measured using 50 mM propanol as substrate at 37ºC. The activity of *Bm*MDH2 incubated in the absence of metal ion was taken as 100%. Data are means ± SDs of three measurements. **b.** The recovery activity of metal ions-free *Bm*MDH2 replenished with various metal ions. The metal ions in *Bm*MDH2 were chelated with EDTA. The activity of *Bm*MDH2 with Mn^2+^ replenishment was taken as 100%. Data are means ± SDs of three measurements.

Fig. S13 The stability of NADx activation effect after incubation in various buffers**.** The purified NADx (NAD^+^ treated with ACT and purified as described in materials and methods) were dissolved in MQ water or various buffer at the concentration of 30 mM and incubated at 37ºC for 30 minutes. Then the samples were used as the cofactor to measure the catalytic rate of *Bm*MDH2 in the presence or absence of ACT at 37ºC. The catalytic rate in the presence of ACT was considered to be fully activated, and was defined as 100%. The reactions were conducted in 200 μL Tris-HCl buffer (100 mM, pH 8.0) containing 40 mM ethanol, 5 mM MgCl_2_, 0.5 mM MnCl_2_, 200 nM *Bm*MDH2, 0 or 100 nM ACT. Data are means ± SDs of three measurements.


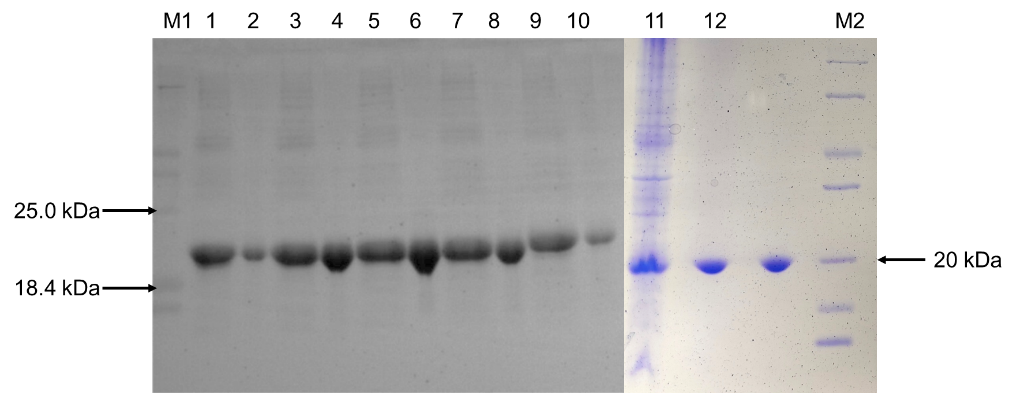


Fig. S14 The SDS-PAGE analysis of purified ACT and its mutants. Lane 1: Crude extracts of ACT_R92A_, Lane 2: Purified ACT_R92A_, Lane 3: Crude extracts of ACT_E93Q_, Lane 4: Purified ACT_E93Q_, Lane 5: Crude extracts of ACT_E96Q_, Lane 6: Purified ACT_E96Q_, Lane 7: Crude extracts of ACT_E97Q_, Lane 8: Purified ACT**_E9_**_7Q_, Lane 9: Crude extracts of ACT_E143Q_, Lane 10: Purified ACT_E143Q_, Lane 11: Crude extracts of ACT_WT_, Lane 12: Purified ACT_WT_.

**References**

[1] A. H. de la Pena, A. Suarez, K. C. Duong-Ly, A. J. Schoeffield, M. A. Pizarro-Dupuy, M. Zarr, S. A. Pineiro, L. M. Amzel, S. B. Gabelli. Structural and enzymatic characterization of a nucleoside diphosphate sugar hydrolase from *Bdellovibrio bacteriovorus*. *PLoS One* **2015**, *10*, e0141716 .

[2] A. S. Mildvan, Z. Xia, H. F. Azurmendi, V. Saraswat, P. M. Legler, M. A. Massiah, S. B. Gabelli, M. A. Bianchet, L. W. Kang, L. M. Amzel. Structures and mechanisms of Nudix hydrolases. *Arch. Biochem. Biophys.* **2005**, *433*, 129-143.
